# Assisted Reproductive Technology Surveillance — United States, 2018

**DOI:** 10.15585/mmwr.ss7104a1

**Published:** 2022-02-18

**Authors:** Saswati Sunderam, Dmitry M. Kissin, Yujia Zhang, Amy Jewett, Sheree L. Boulet, Lee Warner, Charlan D. Kroelinger, Wanda D. Barfield

**Affiliations:** ^1^Division of Reproductive Health, National Center for Chronic Disease Prevention and Health Promotion, CDC; ^2^Emory University, School of Medicine, Atlanta, Georgia

## Abstract

**Problem/Condition:**

Since the first U.S. infant conceived with assisted reproductive technology (ART) was born in 1981, both the use of ART and the number of fertility clinics providing ART services have increased steadily in the United States. ART includes fertility treatments in which eggs or embryos are handled in the laboratory (i.e., in vitro fertilization [IVF] and related procedures). Although the majority of infants conceived through ART are singletons, women who undergo ART procedures are more likely than women who conceive naturally to have multiple births because multiple embryos might be transferred. Multiple births can pose substantial risks for both mothers and infants, including obstetric complications, preterm birth (<37 weeks), and low birthweight (<2,500 g). This report provides state-specific information for the United States (including the District of Columbia and Puerto Rico) on ART procedures performed in 2018 and compares birth outcomes that occurred in 2018 (resulting from ART procedures performed in 2017 and 2018) with outcomes for all infants born in the United States in 2018.

**Period Covered:**

2018.

**Description of System:**

In 1995, CDC began collecting data on ART procedures performed in fertility clinics in the United States as mandated by the Fertility Clinic Success Rate and Certification Act of 1992 (Public Law 102–493 [October 24, 1992]). Data are collected through the National ART Surveillance System (NASS), a web-based data collection system developed by CDC. This report includes data from the 50 U.S. states, the District of Columbia, and Puerto Rico.

**Results:**

In 2018, a total of 203,119 ART procedures (range: 196 in Alaska to 26,028 in California) were performed in 456 U.S. fertility clinics and reported to CDC. These procedures resulted in 73,831 live-birth deliveries (range: 76 in Puerto Rico and Wyoming to 9,666 in California) and 81,478 infants born (range: 84 in Wyoming to 10,620 in California). Nationally, among women aged 15–44 years, the rate of ART procedures performed was 3,135 per 1 million women. ART use exceeded 1.5 times the national rate in seven states (Connecticut, Illinois, Maryland, Massachusetts, New Jersey, New York, and Rhode Island) and the District of Columbia. ART use rates exceeded the national rate in an additional seven states (California, Delaware, Hawaii, New Hampshire, Utah, Vermont, and Virginia).

Nationally, among all ART transfer procedures, the average number of embryos transferred was similar across age groups (1.3 among women aged <35 years, 1.3 among women aged 35–37 years, and 1.4 among women aged >37 years). The national single-embryo transfer (SET) rate among all embryo-transfer procedures was 74.1% among women aged <35 years (range: 28.2% in Puerto Rico to 89.5% in Delaware), 72.8% among women aged 35–37 years (range: 30.6% in Puerto Rico to 93.7% in Delaware), and 66.4% among women aged >37 years (range: 27.1% in Puerto Rico to 85.3% in Delaware).

In 2018, ART contributed to 2.0% of all infants born in the United States (range: 0.4% in Puerto Rico to 5.1% in Massachusetts) from procedures performed in 2017 and 2018. Approximately 78.6% of ART-conceived infants were singleton infants. Overall, ART contributed to 12.5% of all multiple births, including 12.5% of all twin births and 13.3% of all triplets and higher-order births. ART-conceived twins accounted for approximately 97.1% (15,532 of 16,001) of all ART-conceived multiple births. The percentage of multiple births was higher among infants conceived with ART (21.4%) than among all infants born in the total birth population (3.3%). Approximately 20.7% (15,532 of 74,926) of ART-conceived infants were twins, and 0.6% (469 of 74,926) were triplets and higher-order multiples.

Nationally, infants conceived with ART contributed to 4.2% of all low birthweight (<2,500 g) infants. Among ART-conceived infants, 18.3% were low birthweight compared with 8.3% among all infants. ART-conceived infants contributed to 5.1% of all preterm (gestational age <37 weeks) infants. The percentage of preterm births was higher among infants conceived with ART (26.1%) than among all infants born in the total birth population (10.0%).

The percentage of low birthweight among singletons was 8.3% among ART-conceived infants and 6.6% among all infants born. The percentage of preterm births among ART-conceived singleton infants was 14.9% compared with 8.3% among all singleton infants. The percentages of small for gestational age infants was 7.3% among ART-conceived infants compared with 9.4% among all infants.

**Interpretation:**

Although singleton infants accounted for the majority of ART-conceived infants, multiple births from ART varied substantially among states and nationally, contributing to >12% of all twins, triplets, and higher-order multiple infants born in the United States. Because multiple births are associated with higher rates of prematurity than singleton births, the contribution of ART to poor birth outcomes continues to be noteworthy. Although SET rates increased among all age groups, variations in SET rates among states and territories remained, which might reflect variations in embryo-transfer practices among fertility clinics and might in part account for variations in multiple birth rates among states and territories.

**Public Health Action:**

Reducing the number of embryos transferred and increasing use of SET, when clinically appropriate, can help reduce multiple births and related adverse health consequences for both mothers and infants. Whereas risks to mothers from multiple-birth pregnancy include higher rates of caesarean delivery, gestational hypertension, and gestational diabetes, infants from multiple births are at increased risk for numerous adverse sequelae such as preterm birth, birth defects, and developmental disabilities. Long-term follow-up of ART infants through integration of existing maternal and infant health surveillance systems and registries with data available from NASS might be useful for monitoring adverse outcomes on a population basis.

## Introduction

Since the birth of the first U.S. infant conceived with assisted reproductive technology (ART) in 1981, use of advanced technologies to overcome infertility has increased, as has the number of fertility clinics providing ART services and procedures in the United States (*1*). In 1992, Congress passed the Fertility Clinic Success Rate and Certification Act (Public Law 102–493 [October 24, 1992]), which requires that all U.S. fertility clinics performing ART procedures report data to CDC annually on every ART procedure performed. CDC initiated data collection in 1995 and in 1997 published the first annual ART Fertility Clinic Success Rates Report (*2*). The annual ART Fertility Clinic Success Rates Report presents multiple measures of success for ART, including the percentage of ART procedures that result in live-birth deliveries.

Although ART has helped millions of women achieve pregnancy, the treatment is associated with potential health risks for both mothers and infants. Because multiple embryos can be transferred in ART procedures, ART might result in multiple-gestation pregnancies and multiple births (*3*). Obstetric risks to the mothers from a multiple-birth pregnancy include higher rates of caesarean delivery, maternal hemorrhage, pregnancy-related hypertension, and gestational diabetes (*4*–*7*). Risks to the infants include preterm birth, low birthweight, birth defects, developmental disability, and death (*8*–*11*). In addition, singleton infants conceived with ART might have a higher risk for low birthweight and prematurity than singletons not conceived with ART (*12*). However, research published in 2017 suggests that this higher risk might be associated with singleton births resulting from multiple-embryo transfers (*13*).

This report was compiled from data provided and verified by ART clinics on ART procedures performed in 2018 and reported to CDC’s Division of Reproductive Health. Data on the use of ART in 2018 are presented for residents of each U.S. state, the District of Columbia, and Puerto Rico. State-specific data also are reported for outcomes for infants born in 2018 resulting from ART procedures performed in 2017 and 2018. Specifically, the report presents the contribution of ART to selected outcomes (e.g., multiple births, low birthweight, preterm births, and small for gestational age [SGA]) and compares outcomes among ART-conceived infants with outcomes among all infants born in the United States in 2018.

## Methods

### National ART Surveillance System

In 1995, CDC initiated data collection of ART procedures performed in the United States. ART data are obtained from all fertility clinics in the United States that provide and verify information about the outcomes of the ART cycles through the National ART Surveillance System (NASS), a web-based data collection system developed by CDC (https://www.cdc.gov/art/nass/index.html). Clinics that are members of the Society for Assisted Reproductive Technology (SART) can report their data to NASS through SART. Clinics that are not members of SART can enter their data directly in NASS. All clinics must verify the accuracy of the data they report before finalizing submission to NASS. The data then are compiled by a CDC contractor and reviewed for accuracy. In 2018, a total of 8.6% of clinics did not report their data to CDC and are listed as nonreporting clinics in the 2018 ART Fertility Clinic Success Rates Report, as required by the Fertility Clinic Success Rate and Certification Act of 1992. More details about the law are available at https://www.cdc.gov/art/nass/policy.html. Because nonreporting clinics tend to be smaller on average than reporting clinics, NASS is estimated to contain information on 98% of all ART procedures in the United States (*1*).

Data collected include patient demographics, medical history, and infertility diagnoses; clinical information pertaining to the ART procedure type; and information about resultant pregnancies and births. The data file contains one record per ART procedure (i.e., cycle of treatment performed). Because ART providers typically do not provide continued prenatal care after a pregnancy is established, information on live births is collected for all procedures from patients and other physicians. CDC’s NASS data are available to external researchers through the Collaborative for ART Epidemiologic Research (https://www.cdc.gov/art/nass/accessdata.html).

### ART Procedures

ART includes fertility treatments in which eggs or embryos are handled in a laboratory (i.e., in vitro fertilization [IVF], gamete intrafallopian transfer, and zygote intrafallopian transfer). More than 99% of ART procedures performed are IVF. Because an ART procedure consists of multiple steps over an interval of multiple weeks, a procedure often is referred to as a cycle of treatment. An ART cycle usually begins with drug-induced ovarian stimulation. If eggs are produced, the cycle progresses to the egg-retrieval stage, which involves surgical removal of the eggs from the ovaries. After the eggs are retrieved, they are combined with sperm in a laboratory during the IVF procedure. For most IVF procedures (75.7% in 2018), a specialized technique (intracytoplasmic sperm injection) is used in which a single sperm is injected directly into the egg (*1*). If successful fertilization occurs, the most viable embryos (i.e., those that appear morphologically most likely to develop and implant) are selected for transfer back into the uterus. If an embryo implants in the uterus, a clinical pregnancy is diagnosed by the presence of a gestational sac detectable by ultrasound. On average, less than half of the procedures result in a clinical pregnancy. Most pregnancies will progress to a live-birth delivery, defined as the delivery of one or more live-born infants; however, some result in pregnancy loss (*14*,*15*). ART does not include treatments in which only sperm are handled (i.e., intrauterine insemination) or procedures in which a woman is administered drugs to stimulate egg production without the intention of having eggs retrieved.

ART procedures are classified based on the source of the egg (patient or donor) and the status of the eggs and embryos. Both fresh and thawed embryos can be derived from fresh or frozen eggs of the patient or donor. Patient and donor embryos can be created using sperm from a partner or donor. ART procedures involving fresh eggs and embryos include an egg-retrieval stage. ART procedures that use thawed eggs or embryos do not include egg retrieval because the eggs were retrieved during a previous ART procedure, and the eggs were either frozen or fertilized and the resultant embryos were frozen until the current ART procedure. An ART cycle can be discontinued at any step for medical reasons or by patient choice.

### Birth Data for United States

Data on the total numbers of live births, including singleton and multiple births, in each area in 2018 were obtained from U.S. natality data collected via the National Center for Health Statistics (*16*,*17*). The data were derived from birth certificates.

### Variables and Definitions

Data on ART procedures and birth outcomes are presented by patient’s residence (i.e., state or territory) at the time of treatment, which might not be the same as the location where the procedure was performed. If information on a patient’s residence was missing, residence was assigned as the location where the procedure was performed (0.3% of procedures performed in 2018 and 0.1% of live-birth deliveries occurring in 2018). ART procedures performed in the United States among non-U.S. residents are included in NASS data; however, they are excluded from certain calculations because these women might have delivered outside the United States, and the appropriate denominators were not available. To protect confidentiality, table cells with values of 1–4 for ART-conceived infants and 0–9 for all infants are suppressed. The cell suppression criteria for ART population allows for the representation of some clinics that carry out only a small number of cycles while maintaining minimum risks for identification. Because of limited numbers, ART data from U.S. territories (with the exception of Puerto Rico) are not included in this report. In addition, percentages derived from cell values <20 in the denominator have been suppressed because they are unstable.

This report presents data on all procedures performed with the exception of research cycles and cycles in which egg or embryo banking was performed for future ART cycles. The number of ART procedures performed per 1 million women aged 15–44 years was calculated. Data about population size were compiled based on July 1, 2018, estimates from the U.S. Census Bureau (*18*). The resulting rate approximates the proportion of women who used ART in each state or territory. This proxy measure of ART use is only an approximation because certain women who use ART are outside the age range of 15–44 years (approximately 5.4% of cycles performed in 2018), and certain women might have had more than one procedure during the reporting period.

A live-birth delivery was defined as a birth of one or more infants. A singleton live-birth delivery was defined as a delivery of only one infant who was born live. A multiple live-birth delivery was defined as a delivery of two or more infants, at least one of whom was born live. Low birthweight was defined as <2,500 g, moderately low birthweight as 1,500–2,499 g, and very low birthweight as <1,500 g. Gestational age for births among women who did not undergo ART procedures was calculated using obstetric estimate of gestational age at delivery (*19*). For births to women who underwent fresh ART procedures, gestational age was calculated by subtracting the date of egg retrieval from the birth date and adding 14 days. For births to women who underwent frozen embryo cycles or fresh ART procedures for which the date of retrieval was not available, gestational age was calculated by subtracting the date of embryo transfer from the birth date and adding 17 days (to account for an average of 3 days in embryo culture). Preterm birth was defined as gestational age <37 weeks, late preterm 34–36 weeks, early preterm <34 weeks, and very preterm <32 weeks (*17*).

Single-embryo transfer (SET) procedures among all embryo-transfer procedures are reported. In a SET procedure, only one embryo is placed in the uterus per transfer regardless of how many embryos are available. The rate of SET was calculated by dividing the total number of SET procedures by the total number of embryo-transfer procedures performed and reported by the following age groups: <35 years, 35–37 years, and >37 years. The average number of embryos transferred by age group was calculated by dividing the total number of embryos transferred by the total number of embryo-transfer procedures performed among that age group.

The proportion of ART infants among all births in a particular state or territory was used as a second measure of ART use. The proportion of adverse outcomes among ART-conceived infants (e.g., preterm birth) was calculated by dividing the total number of adverse outcomes among ART-conceived infants by the total number of adverse outcomes among all infants born.

The percentage of infants (ART conceived and all infants) born in a state or territory for each plurality group (singleton, multiple, twin, and triplet and higher-order multiple) was calculated by dividing the number of infants (ART conceived and all infants) in each plurality group by the total number of infants born (ART conceived and all infants). The percentage of infants with low birthweight and preterm birth was calculated only for singleton births for ART-conceived infants and for all infants by dividing the number of low birthweight or preterm infants among singletons by the total number of singleton infants.

In addition, the percentage of singleton infants who were SGA (defined as <10th percentile of birthweight for gestational age week and limited to 22–44 weeks) was calculated using a reference distribution (*20*). The percentage of singleton SGA infants was calculated for all births by dividing the number of singleton SGA infants in the gestational age category (week) by the total number of singleton infants in that gestational age category for ART-conceived and all infants, respectively.

To assess the proportion of ART births among U.S. births in 2018, ART births were aggregated from two reporting years: 1) infants conceived with ART procedures performed in 2017 and born in 2018 (71.2% of the live-birth deliveries reported to NASS for 2018) and 2) infants conceived with ART procedures performed in 2018 and born in 2018 (28.8% of the live-birth deliveries reported to NASS for 2018).

## Results

### Overview of Fertility Clinics

**FIGURE 1:**
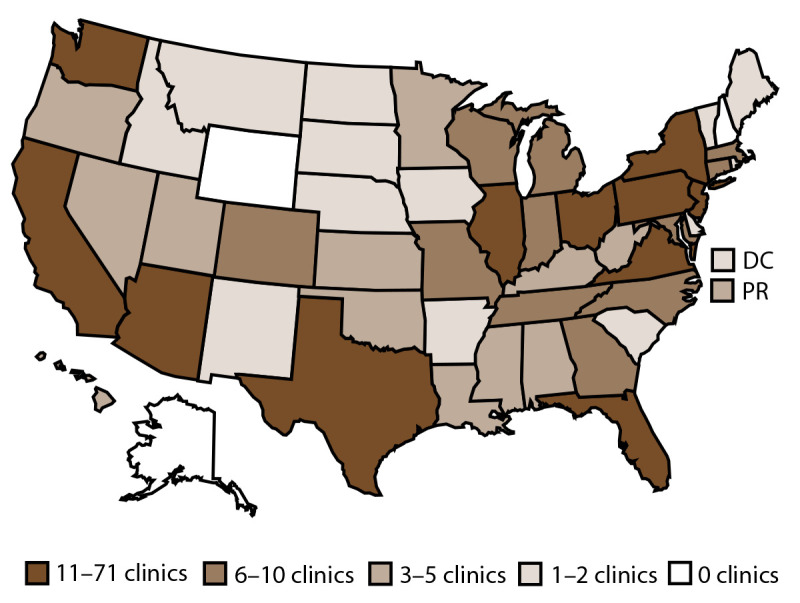
Location and number* of assisted reproductive technology clinics, by quartile — United States and Puerto Rico, 2018 **Abbreviations:** DC = District of Columbia; PR = Puerto Rico. * In 2018, of the 499 clinics in the United States, 456 (91.4%) submitted data.

In 2018, a total of 499 fertility clinics in the United States performed ART procedures and 456 (91.4%) provided data to CDC, with the majority located in or near major cities (*1*). The number of fertility clinics performing ART procedures varied by state or territory. The states with the largest numbers of fertility clinics providing data were California (71), Texas (42), and New York (42) (Figure 1).

### Number and Type of ART Procedures

The number, type, and outcome of ART procedures performed in 2018 are provided according to patient’s residence for all 52 areas and non-U.S. residents (Table 1). Residence data were missing for approximately 0.3% of procedures performed, and in these instances, the patient’s residence was assigned as the location where the ART procedure was performed. In 2018, approximately 14.2% of ART procedures were performed in a state or territory other than the patient’s state or territory of residence. Non-U.S. residents accounted for approximately 3.6% of ART procedures, 4.3% of ART live-birth deliveries, and 4.4% of ART-conceived infants born.

**TABLE 1:** Number and outcomes of assisted reproductive technology procedures performed, by female patient’s area of residence at time of treatment — United States and Puerto Rico, 2018

Patient’s area of residence*	No. of ART clinics^†^	No. of ART procedures performed^§^	No. of ART embryo-transfer procedures^¶^	No. of ART pregnancies	No. of ART live-birth deliveries	No. of ART singleton live-birth deliveries	No. of ART multiple live-birth deliveries	No. of ART live-born infants	No. of ART procedures per 1 million women aged 15–44 yrs**
Alabama	5	1,298	973	471	388	353	35	423	1,368
Alaska	0	196	159	95	80	71	9	89	1,346
Arizona	15	3,160	2,602	1,436	1,189	1,001	188	1,383	2,295
Arkansas	1	586	490	253	210	173	37	249	1,014
California	71	26,028	20,100	11,562	9,666	8,721	945	10,620	3,218
Colorado	8	2,397	2,216	1,479	1,261	1,119	142	1,404	2,063
Connecticut	6	3,558	2,812	1,623	1,343	1,210	133	1,480	5,312
Delaware	2	703	524	319	270	259	11	281	3,911
District of Columbia	2	1,338	1,017	540	438	412	26	465	7,082
Florida	27	9,011	7,146	3,771	3,103	2,765	338	3,447	2,325
Georgia	9	4,527	3,814	2,097	1,706	1,527	179	1,889	2,093
Hawaii	6	1,078	834	495	403	329	74	480	4,075
Idaho	1	611	509	306	272	232	40	312	1,810
Illinois^††^	26	12,884	9,896	5,208	4,217	3,819	398	4,620	5,120
Indiana	8	2,453	1,967	927	779	662	117	899	1,888
Iowa	2	1,560	1,303	778	647	581	66	715	2,622
Kansas	3	970	802	461	383	347	36	420	1,732
Kentucky	4	1,387	1,150	546	453	372	81	536	1,632
Louisiana	5	1,541	1,224	632	502	462	40	542	1,663
Maine	1	493	422	226	192	170	22	215	2,133
Maryland	7	6,551	5,061	2,666	2,071	1,926	145	2,218	5,518
Massachusetts^††^	8	10,390	8,633	4,250	3,469	3,230	239	3,711	7,438
Michigan	10	4,400	3,682	2,018	1,641	1,382	259	1,909	2,338
Minnesota	5	3,088	2,656	1,505	1,289	1,140	149	1,440	2,875
Mississippi	3	628	542	294	243	222	21	265	1,065
Missouri	10	2,447	2,018	1,134	933	796	137	1,071	2,084
Montana	1	289	232	143	114	106	8	123	1,495
Nebraska	2	919	724	407	339	277	62	401	2,467
Nevada	5	1,307	1,038	630	527	442	85	614	2,199
New Hampshire	0	937	791	391	314	279	35	350	3,832
New Jersey^††^	19	9,942	8,547	4,797	3,964	3,727	237	4,206	5,901
New Mexico	2	394	350	207	169	150	19	188	991
New York	42	22,779	18,327	9,020	7,220	6,603	617	7,839	5,832
North Carolina	10	4,331	3,446	2,020	1,665	1,504	161	1,830	2,126
North Dakota	1	245	221	140	118	92	26	143	1,662
Ohio	11	4,988	3,976	2,117	1,749	1,585	164	1,917	2,256
Oklahoma	3	1,025	840	446	376	310	66	440	1,330
Oregon	4	1,332	1,213	822	699	596	103	804	1,623
Pennsylvania	14	7,406	5,888	2,948	2,414	2,245	169	2,585	3,107
Puerto Rico	3	298	258	106	76	55	21	98	484
Rhode Island^††^	1	1,026	865	351	276	245	31	308	4,935
South Carolina	4	1,946	1,510	906	729	652	77	809	1,997
South Dakota	1	304	264	136	119	91	28	147	1,882
Tennessee	10	1,987	1,634	913	767	692	75	843	1,503
Texas	42	15,160	12,529	7,134	5,895	5,225	670	6,572	2,537
Utah	3	2,180	1,833	1,106	914	800	114	1,026	3,158
Vermont	2	435	345	180	148	133	15	163	3,780
Virginia	11	6,252	4,958	2,676	2,194	1,997	197	2,390	3,703
Washington	11	4,171	3,363	2,053	1,707	1,566	141	1,849	2,785
West Virginia	3	409	340	176	142	121	21	165	1,282
Wisconsin	6	2,199	1,818	956	818	719	99	918	2,022
Wyoming	0	209	176	104	76	68	8	84	1,944
Non-U.S. residents	—	7,366	5,851	3,711	3,154	2,729	425	3,583	—^§§^
**Total**	**456**	**203,119**	**163,889**	**89,688**	**73,831**	**66,290**	**7,541**	**81,478**	**3,135**

**Abbreviation:** ART = assisted reproductive technology.

* In cases of missing patient’s residence data (0.3%), the patient’s residence was assigned as the location where the ART procedure was performed.

† The ART procedures and outcomes by patient’s area of residence do not necessarily reflect the procedures and outcomes of the ART clinics within the area because some patients seek treatment at a clinic in a location other than their area of residence.

^§^ The total number of procedures reported to CDC was 306,197. The number of procedures in this report excludes 103,078 cycles in which egg or embryo banking was performed. In addition, all numbers and percentages exclude eight research cycles that were evaluating new procedures.

^¶^ Embryo-transfer procedures include all procedures performed in which at least one embryo was transferred.

** On the basis of U.S. Census Bureau estimates (**Source:** US Census Bureau. Annual estimates of the resident population for selected age groups by sex for the United States, states, counties, and Puerto Rico Commonwealth and municipios: April 1, 2010 to July 1, 2018. Washington, DC: US Census Bureau, Population Division; 2018. https://data.census.gov/cedsci/table?q=United%20States&g=0100000US&tid=ACSST1Y2018.S0101&vintage=2018).

^††^ State with comprehensive insurance mandate requiring insurers to cover the costs associated with diagnosis and treatment of infertility inclusive of ART services for at least four oocyte retrievals.

^§§^ Non-U.S. residents were excluded from rate because the appropriate denominators were not available.

**FIGURE 2:**
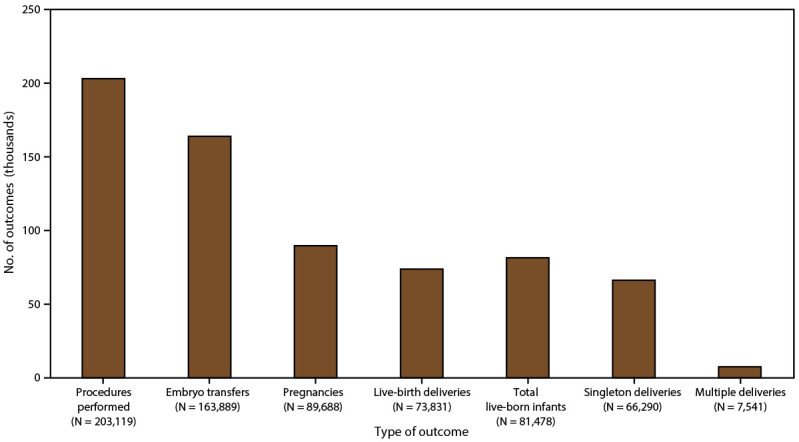
Number and outcomes of assisted reproductive technology procedures performed,* by type of outcome — United States and Puerto Rico, 2018 * The total number of procedures reported to CDC was 306,197. Procedures performed exclude 103,078 cycles in which egg or embryo banking was performed and eight research cycles in which a new treatment procedure was being evaluated. Embryo transfers include all procedures in which at least one embryo was transferred.

In 2018, a total of 306,197 ART procedures were reported to CDC (*1*). This report includes data for 203,119 ART procedures performed (range: 196 in Alaska to 26,028 in California) in the United States (including Puerto Rico) (Table 1) (Figure 2). Excluded are 103,078 cycles in which egg or embryo banking was performed and eight research cycles in which a new treatment procedure was being evaluated. Of 203,119 procedures performed in the 52 states or territories, 163,889 (80.7%) progressed to embryo transfer. Of 163,889 ART procedures that progressed to the embryo-transfer stage, 89,688 (54.7%) resulted in a pregnancy and 73,831 (45.0%) in a live-birth delivery (range: 76 in Puerto Rico and Wyoming to 9,666 in California). The 73,831 live-birth deliveries included 66,290 singleton deliveries (89.8%) and 7,541 multiple deliveries (10.2%) and resulted in 81,478 live-born infants (range: 84 in Wyoming to 10,620 in California).

Six states with the largest numbers of ART procedures (California, Illinois, Massachusetts, New Jersey, New York, and Texas) accounted for approximately half (47.8%; 97,183 of 203,119) of all ART procedures, 47.6% (78,032 of 163,889) of all embryo-transfer procedures, 46.1% (37,568 of 81,478) of all ART-conceived infants born, and 41.2% (3,106 of 7,541) of all ART-conceived multiple live-birth deliveries in the United States (Table 1). However, these six states accounted for only 36.1% of all U.S. births (*17*).

The number of ART procedures per 1 million women aged 15–44 years ranged from 484 in Puerto Rico to 7,438 in Massachusetts, with an overall national rate of 3,135 (Table 1). Fourteen states (California, Connecticut, Delaware, Hawaii, Illinois, Maryland, Massachusetts, New Hampshire, New Jersey, New York, Rhode Island, Utah, Vermont, and Virginia) and the District of Columbia had ART use rates higher than the national rate. The District of Columbia (7,082) and Massachusetts (7,438) had rates exceeding twice the national rate, whereas Connecticut (5,312), Illinois (5,120), Maryland (5,518), New Jersey (5,901), New York (5,832), and Rhode Island (4,935) had rates exceeding 1.5 times the national rate. The three areas with the lowest ART use rates were Arkansas (1,014), New Mexico (991), and Puerto Rico (484).

### Number of Embryos Transferred

Overall, 66,732 embryo-transfer procedures were performed among women aged <35 years, 37,604 among women aged 35–37 years, and 59,553 among women aged >37 years (Table 2). Nationally, on average, 1.3 embryos were transferred per procedure among women aged <35 years, 1.3 embryos among women aged 35–37 years, and 1.4 embryos among women aged >37 years. The national SET rate was 74.1% among women aged <35 years (range: 28.2% in Puerto Rico to 89.5% in Delaware), 72.8% among women aged 35–37 years (range: 30.6% in Puerto Rico to 93.7% in Delaware), and 66.4% among women aged >37 years (range: 27.1% in Puerto Rico to 85.3% in Delaware).

**TABLE 2:** Number of assisted reproductive technology embryo-transfer procedures performed with a single-embryo transfer, by female patient’s age group and area of residence at time of treatment — United States and Puerto Rico, 2018

Patient’s area of residence*	<35 yrs			35–37 yrs			>37 yrs		
	No. of embryo-transfer procedures^†^	Average no. of embryos transferred	SET (%)	No. of embryo-transfer procedures^†^	Average no. of embryos transferred	SET (%)	No. of embryo-transfer procedures^†^	Average no. of embryos transferred	SET (%)
Alabama	513	1.3	71.0	212	1.4	66.0	248	1.5	57.3
Alaska	56	1.3	66.1	39	1.1	87.2	64	1.4	68.8
Arizona	1,161	1.5	57.3	591	1.4	61.8	850	1.5	58.1
Arkansas	262	1.4	59.9	119	1.4	57.1	109	1.6	52.3
California	6,016	1.3	73.9	4,920	1.3	74.5	9,164	1.4	68.6
Colorado	904	1.2	76.8	546	1.2	81.0	766	1.2	77.0
Connecticut	1,109	1.2	80.7	681	1.3	68.4	1,022	1.5	60.9
Delaware	266	1.1	89.5	95	1.1	93.7	163	1.2	85.3
District of Columbia	226	1.1	88.1	229	1.2	83.4	562	1.3	76.0
Florida	2,861	1.3	70.5	1,614	1.3	70.6	2,671	1.4	67.4
Georgia	1,598	1.3	73.2	863	1.3	72.2	1,353	1.3	71.9
Hawaii	268	1.5	51.5	171	1.5	55.6	395	1.7	50.4
Idaho	276	1.4	60.9	76	1.3	68.4	157	1.4	63.7
Illinois^§^	4,105	1.3	72.1	2,406	1.3	67.8	3,385	1.5	59.4
Indiana	1,076	1.4	62.5	439	1.4	62.2	452	1.5	57.5
Iowa	771	1.2	75.7	261	1.3	71.3	271	1.3	72.3
Kansas	420	1.2	82.6	206	1.2	78.2	176	1.3	78.4
Kentucky	662	1.4	63.0	213	1.4	58.7	275	1.6	49.1
Louisiana	636	1.2	82.9	292	1.2	79.1	296	1.3	79.1
Maine	180	1.2	77.8	103	1.3	75.7	139	1.3	74.1
Maryland	1,951	1.2	83.5	1,110	1.2	80.0	2,000	1.4	67.7
Massachusetts^§^	3,319	1.1	87.5	2,138	1.2	81.5	3,176	1.6	60.1
Michigan	1,834	1.5	52.1	825	1.5	54.2	1,023	1.7	52.4
Minnesota	1,311	1.3	68.0	613	1.3	71.9	732	1.4	65.3
Mississippi	316	1.3	74.4	107	1.4	65.4	119	1.2	75.6
Missouri	1,104	1.4	62.0	435	1.5	56.3	479	1.6	58.0
Montana	115	1.2	79.1	61	1.2	82.0	56	1.3	76.8
Nebraska	422	1.3	66.8	149	1.4	60.4	153	1.4	66.0
Nevada	471	1.4	62.2	222	1.3	67.1	345	1.3	73.3
New Hampshire	363	1.2	81.0	200	1.3	74.0	228	1.5	64.5
New Jersey^§^	3,303	1.2	85.4	1,951	1.2	82.6	3,293	1.3	73.9
New Mexico	150	1.3	70.0	94	1.2	77.7	106	1.5	62.3
New York	6,214	1.3	75.2	3,950	1.3	72.2	8,163	1.5	61.9
North Carolina	1,593	1.2	80.7	754	1.3	74.1	1,099	1.4	67.2
North Dakota	111	1.4	55.9	51	1.5	52.9	59	1.4	59.3
Ohio	2,005	1.2	77.6	913	1.3	70.4	1,058	1.4	61.9
Oklahoma	442	1.5	55.0	204	1.6	45.1	194	1.6	47.4
Oregon	438	1.4	64.8	288	1.3	69.8	487	1.3	74.7
Pennsylvania	2,757	1.2	81.8	1,445	1.2	78.2	1,686	1.4	70.3
Puerto Rico	78	1.8	28.2	62	1.8	30.6	118	1.9	27.1
Rhode Island^§^	373	1.2	82.8	209	1.2	76.6	283	1.6	57.6
South Carolina	709	1.3	75.2	345	1.3	66.7	456	1.4	65.6
South Dakota	171	1.6	40.4	50	1.6	42.0	43	1.3	69.8
Tennessee	804	1.2	78.2	347	1.3	70.9	483	1.4	67.5
Texas	5,956	1.3	72.4	2,930	1.3	72.6	3,643	1.4	68.9
Utah	1,064	1.3	69.6	329	1.3	67.5	440	1.4	65.2
Vermont	115	1.3	73.9	75	1.4	62.7	155	1.6	59.4
Virginia	1,836	1.2	79.3	1,175	1.2	78.9	1,947	1.3	72.5
Washington	1,265	1.2	79.4	840	1.2	81.3	1,258	1.3	76.9
West Virginia	193	1.5	58.0	74	1.3	70.3	73	1.5	63.0
Wisconsin	918	1.3	69.9	467	1.4	65.7	433	1.4	63.5
Wyoming	93	1.2	78.5	39	1.3	74.4	44	1.4	63.6
Non-U.S. resident	1,572	1.3	68.0	1,076	1.3	72.1	3,203	1.3	72.7
**Total**	**66,732**	**1.3**	**74.1**	**37,604**	**1.3**	**72.8**	**59,553**	**1.4**	**66.4**

**Abbreviation:** SET = single-embryo transfer.

* In cases of missing patient’s residence data (0.3%), the patient’s residence was assigned as the location where the assisted reproductive technology procedure was performed.

^†^ Includes all procedures in which at least one embryo was transferred.

^§^ State with comprehensive insurance mandate requiring insurers to cover the costs associated with diagnosis and treatment of infertility inclusive of ART services for at least four oocyte retrievals.

### Singleton and Multiple Births

To allow comparisons between ART infants and all infants born in the United States, this report includes all ART infants born in 2018. These infants resulted from ART procedures performed in 2017 or 2018.

In 2018, among 3,813,136 infants born in the United States and Puerto Rico, 74,926 (2.0%) were conceived with ART procedures performed in 2017 and 2018 (Table 3). California, New York, and Texas had the highest total numbers of all infants born (454,920; 226,238; and 378,624, respectively) and ART-conceived infants born (10,016; 7,742; and 6,173, respectively). The percentage of ART-conceived infants among all infants born was highest in Massachusetts (5.1%), followed by the District of Columbia (4.6%) and New Jersey (4.2%).

**TABLE 3:** Number, proportion, and percentage of infants born with use of assisted reproductive technology, by female patient’s area of residence at time of treatment — United States and Puerto Rico, 2018

Patient’s area of residence*	Total infants born^†,§^	ART infants born^¶^	Proportion of ART infants among all infants	Singleton infants among ART infants	Singleton infants among all infants	Proportion of ART singleton infants among all singleton infants
	No.	No.	%	No. (%)	No. (%)	%
Alabama	57,761	389	0.7	284 (73.0)	55,655 (96.4)	0.5
Alaska	10,086	76	0.8	56 (73.7)	9,741 (96.6)	0.6
Arizona	80,723	1,329	1.6	898 (67.6)	78,082 (96.7)	1.2
Arkansas	37,018	246	0.7	149 (60.6)	35,854 (96.9)	0.4
California	454,920	10,016	2.2	8,046 (80.3)	440,699 (96.9)	1.8
Colorado	62,885	1,418	2.3	1,097 (77.4)	60,867 (96.8)	1.8
Connecticut	34,725	1,426	4.1	1,133 (79.5)	33,429 (96.3)	3.4
Delaware	10,621	229	2.2	215 (93.9)	10,323 (97.2)	2.1
District of Columbia	9,212	428	4.6	380 (88.8)	8,887 (96.5)	4.3
Florida	221,542	3,236	1.5	2,450 (75.7)	214,480 (96.8)	1.1
Georgia	126,172	1,843	1.5	1,422 (77.2)	121,778 (96.5)	1.2
Hawaii	16,972	425	2.5	274 (64.5)	16,443 (96.9)	1.7
Idaho	21,403	312	1.5	203 (65.1)	20,690 (96.7)	1.0
Illinois**	144,815	4,474	3.1	3,591 (80.3)	139,561 (96.4)	2.6
Indiana	81,646	831	1.0	598 (72.0)	78,876 (96.6)	0.8
Iowa	37,785	689	1.8	543 (78.8)	36,433 (96.4)	1.5
Kansas	36,261	401	1.1	333 (83.0)	35,097 (96.8)	0.9
Kentucky	53,922	533	1.0	369 (69.2)	52,091 (96.6)	0.7
Louisiana	59,615	539	0.9	430 (79.8)	57,526 (96.5)	0.7
Maine	12,311	206	1.7	152 (73.8)	11,884 (96.5)	1.3
Maryland	71,080	2,192	3.1	1,840 (83.9)	68,592 (96.5)	2.7
Massachusetts**	69,109	3,543	5.1	2,996 (84.6)	66,664 (96.5)	4.5
Michigan	110,032	1,778	1.6	1,194 (67.2)	105,973 (96.3)	1.1
Minnesota	67,344	1,396	2.1	1,047 (75.0)	65,015 (96.5)	1.6
Mississippi	37,000	244	0.7	196 (80.3)	35,697 (96.5)	0.5
Missouri	73,269	968	1.3	670 (69.2)	70,611 (96.4)	0.9
Montana	11,513	133	1.2	110 (82.7)	11,184 (97.1)	1.0
Nebraska	25,488	347	1.4	239 (68.9)	24,551 (96.3)	1.0
Nevada	35,682	567	1.6	421 (74.3)	34,638 (97.1)	1.2
New Hampshire	11,995	438	3.7	328 (74.9)	11,579 (96.5)	2.8
New Jersey**	101,223	4,239	4.2	3,625 (85.5)	97,667 (96.5)	3.7
New Mexico	23,039	186	0.8	148 (79.6)	22,453 (97.5)	0.7
New York	226,238	7,742	3.4	6,266 (80.9)	218,244 (96.5)	2.9
North Carolina	118,954	1,855	1.6	1,436 (77.4)	114,948 (96.6)	1.2
North Dakota	10,636	133	1.3	90 (67.7)	10,290 (96.7)	0.9
Ohio	135,134	1,840	1.4	1,483 (80.6)	130,554 (96.6)	1.1
Oklahoma	49,800	393	0.8	271 (69.0)	48,129 (96.6)	0.6
Oregon	42,188	756	1.8	534 (70.6)	40,698 (96.5)	1.3
Pennsylvania	135,673	2,538	1.9	2,142 (84.4)	131,199 (96.7)	1.6
Puerto Rico	21,424	84	0.4	46 (54.8)	20,977 (97.9)	0.2
Rhode Island**	10,506	301	2.9	240 (79.7)	10,123 (96.4)	2.4
South Carolina	56,669	692	1.2	535 (77.3)	54,785 (96.7)	1.0
South Dakota	11,893	129	1.1	91 (70.5)	11,466 (96.4)	0.8
Tennessee	80,751	794	1.0	601 (75.7)	78,042 (96.6)	0.8
Texas	378,624	6,173	1.6	4,704 (76.2)	366,664 (96.8)	1.3
Utah	47,209	1,091	2.3	786 (72.0)	45,580 (96.5)	1.7
Vermont	5,432	120	2.2	91 (75.8)	5,249 (96.6)	1.7
Virginia	99,843	2,339	2.3	1,946 (83.2)	96,370 (96.5)	2.0
Washington	86,085	1,733	2.0	1,408 (81.2)	83,506 (97.0)	1.7
West Virginia	18,248	121	0.7	87 (71.9)	17,657 (96.8)	0.5
Wisconsin	64,098	917	1.4	665 (72.5)	61,795 (96.4)	1.1
Wyoming	6,562	98	1.5	66 (67.3)	6,332 (96.5)	1.0
**Total**	**3,813,136**	**74,926**	**2.0**	**58,925 (78.6)**	**3,685,628 (96.7)**	**1.6**

**Abbreviation:** ART = assisted reproductive technology.

* In cases of missing patient’s residence data (0.3%), the patient’s residence was assigned as the location where the ART procedure was performed.

^†^ U.S. births exclude births to non-U.S. residents** (Source:** Martin JA, Hamilton BE, Osterman MJ, Driscoll AK. Births: final data for 2018. Natl Vital Stat Rep 2018;68:1–47).

^§^ U.S. births exclude births to non-U.S. residents (**Source:** National Center for Health Statistics, Vital statistics data available. Natality public use file and CD-ROM. Hyattsville, MD, National Center for Health Statistics).

^¶^ Includes infants conceived from ART procedures performed in 2017 and born in 2018 and infants conceived from ART procedures performed in 2018 and born in 2018. Total ART births exclude births to non-U.S. residents.

** State with comprehensive insurance mandate requiring insurers to cover the costs associated with diagnosis and treatment of infertility inclusive of ART services for at least four oocyte retrievals.

Nationally, 21.4% of ART-conceived infants were born in multiple-birth deliveries (range: 6.1% in Delaware to 45.2% in Puerto Rico), compared with 3.3% of all infants (range: 2.1% in Puerto Rico to 3.7% in Connecticut, Michigan, and Nebraska) (Table 4). ART-conceived twins accounted for approximately 97.1% (15,532 of 16,001) of all ART-conceived infants born in multiple-birth deliveries. ART-conceived multiple births contributed to 12.5% of all multiple births (range: 3.7% in Mississippi to 28.5% in Hawaii). Approximately 20.7% of all ART-conceived infants were twins, compared with 3.3% of all infants. ART-conceived twins contributed to 12.5% of all twins. Of ART-conceived infants, 0.6% were triplets and higher-order multiples, compared with 0.1% among all infants. ART-conceived triplets and higher-order multiples contributed to 13.3% of all triplets and higher-order multiples.

**TABLE 4:** Number, percentage, and proportion of multiple-birth infants, twins, and triplets and higher-order infants born with use of assisted reproductive technology procedures, by female patient’s area of residence at time of treatment — United States and Puerto Rico, 2018

Patient’s area of residence*	Multiple-birth infants among ART infants^†,§^	Multiple births among all infants^¶^	Proportion of ART multiple births among all multiple births	Twin infants among ART infants^†,§^	Twin infants among all infants^¶^	Proportion of ART twin infants among all twin infants	Triplets and higher-order infants among ART infants^†,§^	Triplets and higher-order infants among all infants^¶^	Proportion of ART triplets and higher-order infants among all triplets and higher-order infants
	No. (%)	No. (%)	%	No. (%)	No. (%)	%	No. (%)	No. (%)	%
Alabama	105 (27.0)	2,106 (3.6)	5.0	96 (24.7)	2,015 (3.5)	4.8	9 (2.3)	91 (0.2)	9.9
Alaska	20 (26.3)	345 (3.4)	5.8	20 (26.3)	330 (3.3)	6.1	0 (0.0)	15 (0.1)	—^††^
Arizona	431 (32.4)	2,641 (3.3)	16.3	412 (31.0)	2,545 (3.2)	16.2	19 (1.4)	96 (0.1)	19.8
Arkansas	97 (39.4)	1,164 (3.1)	8.3	88 (35.8)	1,124 (3.0)	7.8	9 (3.7)	40 (0.1)	22.5
California	1,970 (19.7)	14,221 (3.1)	13.9	1,928 (19.2)	13,851 (3.0)	13.9	42 (0.4)	370 (0.1)	11.4
Colorado	321 (22.6)	2,018 (3.2)	15.9	321 (22.6)	1,990 (3.2)	16.1	0 (0.0)	28 (0.0)	0.0
Connecticut	293 (20.5)	1,296 (3.7)	22.6	287 (20.1)	1,265 (3.6)	22.7	6 (0.4)	31 (0.1)	19.4
Delaware	14 (6.1)	298 (2.8)	4.7	14 (6.1)	—**	—**	0 (0.0)	—**	—**^,††^
District of Columbia	48 (11.2)	325 (3.5)	14.8	48 (11.2)	314 (3.4)	15.3	0 (0.0)	11 (0.1)	—^††^
Florida	786 (24.3)	7,062 (3.2)	11.1	759 (23.5)	6,906 (3.1)	11.0	27 (0.8)	156 (0.1)	17.3
Georgia	421 (22.8)	4,394 (3.5)	9.6	415 (22.5)	4,281 (3.4)	9.7	6 (0.3)	113 (0.1)	5.3
Hawaii	151 (35.5)	529 (3.1)	28.5	142 (33.4)	511 (3.0)	27.8	9 (2.1)	18 (0.1)	—^††^
Idaho	109 (34.9)	713 (3.3)	15.3	109 (34.9)	701 (3.3)	15.5	0 (0.0)	12 (0.1)	—^††^
Illinois	883 (19.7)	5,254 (3.6)	16.8	862 (19.3)	5,102 (3.5)	16.9	21 (0.5)	152 (0.1)	13.8
Indiana	233 (28.0)	2,770 (3.4)	8.4	227 (27.3)	2,699 (3.3)	8.4	6 (0.7)	71 (0.1)	8.5
Iowa	146 (21.2)	1,352 (3.6)	10.8	140 (20.3)	1,320 (3.5)	10.6	6 (0.9)	32 (0.1)	18.8
Kansas	68 (17.0)	1,164 (3.2)	5.8	68 (17.0)	1,152 (3.2)	5.9	0 (0.0)	12 (0.0)	—^††^
Kentucky	164 (30.8)	1,831 (3.4)	9.0	153 (28.7)	1,799 (3.3)	8.5	11 (2.1)	32 (0.1)	34.4
Louisiana	109 (20.2)	2,089 (3.5)	5.2	—**	2,029 (3.4)	—**	—**	60 (0.1)	—**
Maine	54 (26.2)	427 (3.5)	12.6	48 (23.3)	408 (3.3)	11.8	6 (2.9)	19 (0.2)	—^††^
Maryland	352 (16.1)	2,488 (3.5)	14.1	340 (15.5)	2,397 (3.4)	14.2	12 (0.5)	91 (0.1)	13.2
Massachusetts	547 (15.4)	2,445 (3.5)	22.4	523 (14.8)	2,373 (3.4)	22.0	24 (0.7)	72 (0.1)	33.3
Michigan	584 (32.8)	4,059 (3.7)	14.4	560 (31.5)	3,950 (3.6)	14.2	24 (1.3)	109 (0.1)	22.0
Minnesota	349 (25.0)	2,329 (3.5)	15.0	336 (24.1)	2,270 (3.4)	14.8	13 (0.9)	59 (0.1)	22.0
Mississippi	48 (19.7)	1,303 (3.5)	3.7	42 (17.2)	1,251 (3.4)	3.4	6 (2.5)	52 (0.1)	11.5
Missouri	298 (30.8)	2,658 (3.6)	11.2	285 (29.4)	2,593 (3.5)	11.0	13 (1.3)	65 (0.1)	20.0
Montana	23 (17.3)	329 (2.9)	7.0	—**	—**	—**	—**	—**	—**
Nebraska	108 (31.1)	937 (3.7)	11.5	—**	894 (3.5)	—**	—**	43 (0.2)	—**
Nevada	146 (25.7)	1,044 (2.9)	14.0	140 (24.7)	1,026 (2.9)	13.6	6 (1.1)	18 (0.1)	—^††^
New Hampshire	110 (25.1)	416 (3.5)	26.4	110 (25.1)	416 (3.5)	26.4	0 (0.0)	0 (0.0)	—^††^
New Jersey	614 (14.5)	3,556 (3.5)	17.3	599 (14.1)	3,457 (3.4)	17.3	15 (0.4)	99 (0.1)	15.2
New Mexico	38 (20.4)	586 (2.5)	6.5	38 (20.4)	573 (2.5)	6.6	0 (0.0)	13 (0.1)	—^††^
New York	1,476 (19.1)	7,994 (3.5)	18.5	1,440 (18.6)	7,754 (3.4)	18.6	36 (0.5)	240 (0.1)	15.0
North Carolina	419 (22.6)	4,006 (3.4)	10.5	404 (21.8)	3,873 (3.3)	10.4	15 (0.8)	133 (0.1)	11.3
North Dakota	43 (32.3)	346 (3.3)	12.4	43 (32.3)	—**	—**	0 (0.0)	—**	—**
Ohio	357 (19.4)	4,580 (3.4)	7.8	344 (18.7)	4,431 (3.3)	7.8	13 (0.7)	149 (0.1)	8.7
Oklahoma	122 (31.0)	1,671 (3.4)	7.3	122 (31.0)	1,642 (3.3)	7.4	0 (0.0)	29 (0.1)	0.0
Oregon	222 (29.4)	1,490 (3.5)	14.9	210 (27.8)	1,464 (3.5)	14.3	12 (1.6)	26 (0.1)	46.2
Pennsylvania	396 (15.6)	4,474 (3.3)	8.9	374 (14.7)	4,338 (3.2)	8.6	22 (0.9)	136 (0.1)	16.2
Puerto Rico	38 (45.2)	447 (2.1)	8.5	38 (45.2)	—**	—**	0 (0.0)	—**	—**
Rhode Island	61 (20.3)	383 (3.6)	15.9	—**	365 (3.5)	—**	—**	18 (0.2)	—**^,††^
South Carolina	157 (22.7)	1,884 (3.3)	8.3	157 (22.7)	1,860 (3.3)	8.4	0 (0.0)	24 (0.0)	0.0
South Dakota	38 (29.5)	427 (3.6)	8.9	38 (29.5)	—**	—**	0 (0.0)	—**	—**
Tennessee	193 (24.3)	2,709 (3.4)	7.1	181 (22.8)	2,624 (3.2)	6.9	12 (1.5)	85 (0.1)	14.1
Texas	1,469 (23.8)	11,960 (3.2)	12.3	1,445 (23.4)	11,603 (3.1)	12.5	24 (0.4)	357 (0.1)	6.7
Utah	305 (28.0)	1,629 (3.5)	18.7	299 (27.4)	1,571 (3.3)	19.0	6 (0.5)	58 (0.1)	10.3
Vermont	29 (24.2)	183 (3.4)	15.8	29 (24.2)	—**	—**	0 (0.0)	—**	—**
Virginia	393 (16.8)	3,473 (3.5)	11.3	381 (16.3)	3,368 (3.4)	11.3	12 (0.5)	105 (0.1)	11.4
Washington	325 (18.8)	2,579 (3.0)	12.6	—**	2,501 (2.9)	—**	—**	78 (0.1)	—**
West Virginia	34 (28.1)	591 (3.2)	5.8	34 (28.1)	—**	—**	0 (0.0)	—**	—**
Wisconsin	252 (27.5)	2,303 (3.6)	10.9	240 (26.2)	2,236 (3.5)	10.7	12 (1.3)	67 (0.1)	17.9
Wyoming	32 (32.7)	230 (3.5)	13.9	32 (32.7)	220 (3.4)	14.5	0 (0.0)	10 (0.2)	—^††^
**Total**	**16,001 (21.4)**	**127,508 (3.3)**	**12.5**	**15,532 (20.7)**	**123,974 (3.3)**	**12.5**	**469 (0.6)**	**3,534 (0.1)**	**13.3**

**Abbreviation:** ART = assisted reproductive technology.

* In cases of missing patient’s residence data (0.3%), the patient’s residence was assigned as the location where the ART procedure was performed.

^†^ ART totals include infants conceived from ART procedures performed in 2017 and born in 2018 and infants conceived from ART procedures performed in 2018 and born in 2018. Total ART births exclude births to non-U.S. residents.

^§^ Includes only the number of infants live born in a multiple-birth delivery. For example, if three infants were born in a live-birth delivery and one of the three infants was stillborn, the total number of live-born infants would be two. However, the two infants still would be counted as triplets.

^¶^ U.S. births exclude births to non-U.S. residents (**Source:** National Center for Health Statistics, Vital statistics data available. Natality public use file and CD-ROM. Hyattsville, MD, National Center for Health Statistics).

** To protect confidentiality, cells with values of 1–4 for ART infants and cells with values of 0–9 for all infants are suppressed. Also suppressed are data that can be used to derive suppressed cell values. These values are included in the totals.

^††^ Estimates on the basis of N <20 in the denominator have been suppressed because such rates are considered unstable.

### Adverse Perinatal Outcomes

Nationally, ART-conceived infants contributed to 4.2% of all infants with low birthweight, 4.2% of all infants with moderately low birthweight, and 4.1% of all infants with very low birthweight (Table 5). Among all ART-conceived infants (including multiples), 18.3% had low birthweight compared with 8.3% among all infants (including multiples). Approximately 3.0% of all ART-conceived infants (including multiples) had very low birthweight compared with 1.4% among all infants (including multiples).

**TABLE 5:** Number, percentage, and proportion of infants born with use of assisted reproductive technology, by low birthweight category and female patient’s area of residence at time of treatment — United States and Puerto Rico, 2018

Patient’s area of residence*	<1,500 g (VLBW)			1,500–2,499 g (MLBW)			<2,500 g (LBW)		
	ART infants^†^	All infants^§^	Proportion of ART infants among all infants	ART infants^†^	All infants^§^	Proportion of ART infants among all infants	ART infants^†^	All infants^§^	Proportion of ART infants among all infants
	No. (%)	No. (%)	%	No. (%)	No. (%)	%	No. (%)	No. (%)	%
Alabama	21 (5.7)	1,084 (1.9)	1.9	52 (14.1)	5,100 (8.8)	1.0	73 (19.8)	6,184 (10.7)	1.2
Alaska	—^¶^	98 (1.0)	—^¶^	—^¶^	492 (4.9)	—^¶^	15 (20.0)	590 (5.8)	2.5
Arizona	45 (3.5)	977 (1.2)	4.6	278 (21.8)	5,139 (6.4)	5.4	323 (25.3)	6,116 (7.6)	5.3
Arkansas	6 (2.4)	565 (1.5)	1.1	59 (24.0)	2,900 (7.8)	2.0	65 (26.4)	3,465 (9.4)	1.9
California	212 (2.2)	5,039 (1.1)	4.2	1,378 (14.2)	26,706 (5.9)	5.2	1,590 (16.4)	31,745 (7.0)	5.0
Colorado	26 (1.9)	761 (1.2)	3.4	260 (19.2)	5,145 (8.2)	5.1	286 (21.1)	5,906 (9.4)	4.8
Connecticut	34 (2.4)	459 (1.3)	7.4	208 (14.7)	2,182 (6.3)	9.5	242 (17.1)	2,641 (7.6)	9.2
Delaware	8 (3.6)	161 (1.5)	5.0	21 (9.5)	787 (7.4)	2.7	29 (13.1)	948 (8.9)	3.1
District of Columbia	—^¶^	165 (1.8)	—^¶^	—^¶^	753 (8.2)	—^¶^	43 (10.2)	918 (10.0)	4.7
Florida	137 (4.3)	3,482 (1.6)	3.9	527 (16.7)	15,735 (7.1)	3.3	664 (21.0)	19,217 (8.7)	3.5
Georgia	46 (2.6)	2,259 (1.8)	2.0	306 (17.5)	10,474 (8.3)	2.9	352 (20.1)	12,733 (10.1)	2.8
Hawaii	21 (5.2)	240 (1.4)	8.8	92 (22.9)	1,176 (6.9)	7.8	113 (28.1)	1,416 (8.3)	8.0
Idaho	19 (6.2)	224 (1.0)	8.5	69 (22.5)	1,329 (6.2)	5.2	88 (28.7)	1,553 (7.3)	5.7
Illinois	116 (2.7)	2,132 (1.5)	5.4	647 (14.8)	10,238 (7.1)	6.3	763 (17.4)	12,370 (8.5)	6.2
Indiana	29 (3.6)	1,111 (1.4)	2.6	156 (19.3)	5,461 (6.7)	2.9	185 (22.9)	6,572 (8.0)	2.8
Iowa	21 (3.1)	425 (1.1)	4.9	107 (15.7)	2,183 (5.8)	4.9	128 (18.7)	2,608 (6.9)	4.9
Kansas	8 (2.1)	459 (1.3)	1.7	55 (14.7)	2,217 (6.1)	2.5	63 (16.8)	2,676 (7.4)	2.4
Kentucky	16 (3.1)	756 (1.4)	2.1	95 (18.4)	4,026 (7.5)	2.4	111 (21.6)	4,782 (8.9)	2.3
Louisiana	17 (3.2)	1,145 (1.9)	1.5	85 (16.1)	5,283 (8.9)	1.6	102 (19.4)	6,428 (10.8)	1.6
Maine	—^¶^	121 (1.0)	—^¶^	—^¶^	764 (6.2)	—^¶^	30 (15.2)	885 (7.2)	3.4
Maryland	73 (3.4)	1,175 (1.7)	6.2	272 (12.5)	5,091 (7.2)	5.3	345 (15.9)	6,266 (8.8)	5.5
Massachusetts	71 (2.1)	822 (1.2)	8.6	454 (13.1)	4,415 (6.4)	10.3	525 (15.2)	5,237 (7.6)	10.0
Michigan	65 (3.8)	1,485 (1.3)	4.4	325 (18.8)	7,817 (7.1)	4.2	390 (22.5)	9,302 (8.5)	4.2
Minnesota	51 (3.7)	786 (1.2)	6.5	227 (16.4)	3,831 (5.7)	5.9	278 (20.1)	4,617 (6.9)	6.0
Mississippi	11 (4.6)	794 (2.1)	1.4	45 (18.9)	3,690 (10.0)	1.2	56 (23.5)	4,484 (12.1)	1.2
Missouri	28 (3.1)	991 (1.4)	2.8	184 (20.1)	5,398 (7.4)	3.4	212 (23.2)	6,389 (8.7)	3.3
Montana	—^¶^	108 (0.9)	—^¶^	—^¶^	747 (6.5)	—^¶^	24 (18.3)	855 (7.4)	2.8
Nebraska	8 (2.4)	287 (1.1)	2.8	69 (20.5)	1,640 (6.4)	4.2	77 (22.8)	1,927 (7.6)	4.0
Nevada	33 (6.1)	459 (1.3)	7.2	102 (19.0)	2,638 (7.4)	3.9	135 (25.1)	3,097 (8.7)	4.4
New Hampshire	12 (2.8)	131 (1.1)	9.2	67 (15.5)	681 (5.7)	9.8	79 (18.2)	812 (6.8)	9.7
New Jersey	102 (2.5)	1,401 (1.4)	7.3	512 (12.3)	6,600 (6.5)	7.8	614 (14.8)	8,001 (7.9)	7.7
New Mexico	—^¶^	294 (1.3)	—^¶^	—^¶^	1,790 (7.8)	—^¶^	48 (25.9)	2,084 (9.0)	2.3
New York	212 (2.9)	2,968 (1.3)	7.1	1,001 (13.7)	15,240 (6.7)	6.6	1,213 (16.6)	18,208 (8.0)	6.7
North Carolina	63 (3.7)	1,887 (1.6)	3.3	250 (14.7)	9,083 (7.6)	2.8	313 (18.4)	10,970 (9.2)	2.9
North Dakota	11 (8.7)	121 (1.1)	9.1	22 (17.5)	577 (5.4)	3.8	33 (26.2)	698 (6.6)	4.7
Ohio	47 (2.7)	1,995 (1.5)	2.4	253 (14.5)	9,476 (7.0)	2.7	300 (17.2)	11,471 (8.5)	2.6
Oklahoma	11 (2.8)	721 (1.4)	1.5	81 (20.8)	3,394 (6.8)	2.4	92 (23.7)	4,115 (8.3)	2.2
Oregon	31 (4.2)	370 (0.9)	8.4	120 (16.3)	2,456 (5.8)	4.9	151 (20.5)	2,826 (6.7)	5.3
Pennsylvania	66 (2.7)	1,869 (1.4)	3.5	308 (12.7)	9,353 (6.9)	3.3	374 (15.4)	11,222 (8.3)	3.3
Puerto Rico	9 (10.7)	302 (1.4)	3.0	26 (31.0)	1,910 (8.9)	1.4	35 (41.7)	2,212 (10.3)	1.6
Rhode Island	15 (5.2)	136 (1.3)	11.0	41 (14.2)	665 (6.3)	6.2	56 (19.4)	801 (7.6)	7.0
South Carolina	20 (3.1)	965 (1.7)	2.1	112 (17.3)	4,467 (7.9)	2.5	132 (20.3)	5,432 (9.6)	2.4
South Dakota	10 (7.9)	104 (0.9)	9.6	22 (17.5)	685 (5.8)	3.2	32 (25.4)	789 (6.6)	4.1
Tennessee	34 (4.4)	1,303 (1.6)	2.6	126 (16.5)	6,168 (7.6)	2.0	160 (20.9)	7,471 (9.3)	2.1
Texas	211 (3.5)	5,345 (1.4)	3.9	1,021 (17.1)	26,692 (7.0)	3.8	1,232 (20.6)	32,037 (8.5)	3.8
Utah	44 (4.2)	495 (1.0)	8.9	190 (18.1)	2,890 (6.1)	6.6	234 (22.2)	3,385 (7.2)	6.9
Vermont	—^¶^	58 (1.1)	—^¶^	—^¶^	323 (5.9)	—^¶^	17 (14.9)	381 (7.0)	4.5
Virginia	67 (2.9)	1,453 (1.5)	4.6	275 (12.1)	6,722 (6.7)	4.1	342 (15.0)	8,175 (8.2)	4.2
Washington	30 (1.8)	865 (1.0)	3.5	261 (15.3)	4,825 (5.6)	5.4	291 (17.1)	5,690 (6.6)	5.1
West Virginia	—^¶^	285 (1.6)	—^¶^	—^¶^	1,423 (7.8)	—^¶^	24 (20.9)	1,708 (9.4)	1.4
Wisconsin	19 (2.1)	765 (1.2)	2.5	158 (17.8)	4,170 (6.5)	3.8	177 (19.9)	4,935 (7.7)	3.6
Wyoming	—^¶^	70 (1.1)	—^¶^	—^¶^	544 (8.3)	—^¶^	25 (25.8)	614 (9.4)	4.1
**Total**	**2,158 (3.0)**	**52,473 (1.4)**	**4.1**	**11,123 (15.4)**	**263,491 (6.9)**	**4.2**	**13,281 (18.3)**	**315,964 (8.3)**	**4.2**

**Abbreviations:** ART = assisted reproductive technology; LBW = low birthweight; MLBW = moderately low birthweight; VLBW = very low birthweight.

* In cases of missing patient’s residence data (0.3%), the patient’s residence was assigned as the location where the ART procedure was performed.

^† ^ART totals include infants conceived from ART procedures performed in 2017 and born in 2018 and infants conceived from ART procedures performed in 2018 and born in 2018. Total ART infants exclude births to non-U.S. residents and include only infants with birthweight data available.

^§^ U.S. births exclude births to non-U.S. residents (**Source:** National Center for Health Statistics, Vital statistics data available. Natality public use file and CD-ROM. Hyattsville, MD, National Center for Health Statistics).

^¶^ To protect confidentiality, cells with values of 1–4 for ART infants and cells with values of 0–9 for all infants are suppressed. Also suppressed are data that can be used to derive suppressed cell values. These values are included in the totals.

Nationally, ART contributed to approximately 5.1% of all infants born preterm, 5.0% very preterm, 5.5% early preterm, and 4.9% late preterm (Table 6). In Connecticut and Massachusetts, the contribution of ART to preterm infants was ≥10%. Among all ART-conceived infants (including multiples), 26.1% had born preterm, 4.0% very preterm, 7.8% early preterm, and 18.4% late preterm. Corresponding rates among all infants (including multiples) were 10.0% preterm, 1.6% very preterm, 2.8% early preterm, and 7.3% late preterm. Late preterm births accounted for the majority of preterm births among both ART-conceived infants and all infants (70.2% and 72.6%, respectively).

**TABLE 6:** Number, percentage, and proportion of infants born with use of assisted reproductive technology, by preterm gestational age category and female patient’s area of residence at time of treatment — United States and Puerto Rico, 2018

Patient’s area of residence*	VPTB (<32 wks)			Early PTB (<34 wks)			Late PTB (34–36 wks)			PTB (<37 wks)		
	ART infants^†^	All infants^§^	Proportion of ART infants among all infants	ART infants^†^	All infants^§^	Proportion of ART infants among all infants	ART infants^†^	All infants^§^	Proportion of ART infants among all infants	ART infants^†^	All infants^§^	Proportion of ART infants among all infants
	No. (%)	No. (%)	%	No. (%)	No. (%)	%	No. (%)	No. (%)	%	No. (%)	No. (%)	%
Alabama	32 (8.3)	1,194 (2.1)	2.7	46 (11.9)	2,088 (3.6)	2.2	72 (18.6)	5,116 (8.9)	1.4	118 (30.5)	7,204 (12.5)	1.6
Alaska	—^¶^ (1.4)	131 (1.3)	0.8	—^¶^	236 (2.3)	—^¶^	—^¶^	697 (6.9)	—^¶^	12 (16.4)	933 (9.3)	1.3
Arizona	67 (5.0)	1,127 (1.4)	5.9	135 (10.2)	1,968 (2.4)	6.9	330 (24.8)	5,705 (7.1)	5.8	465 (35.0)	7,673 (9.5)	6.1
Arkansas	10 (4.1)	644 (1.7)	1.6	15 (6.1)	1,154 (3.1)	1.3	64 (26.1)	3,145 (8.5)	2.0	79 (32.2)	4,299 (11.6)	1.8
California	324 (3.3)	5,888 (1.3)	5.5	667 (6.7)	10,438 (2.3)	6.4	1,620 (16.3)	29,526 (6.5)	5.5	2,287 (23.0)	39,964 (8.8)	5.7
Colorado	40 (2.8)	887 (1.4)	4.5	100 (7.1)	1,551 (2.5)	6.4	305 (21.7)	4,244 (6.7)	7.2	405 (28.8)	5,795 (9.2)	7.0
Connecticut	40 (2.8)	517 (1.5)	7.7	112 (7.9)	905 (2.6)	12.4	213 (15.0)	2,346 (6.8)	9.1	325 (22.9)	3,251 (9.4)	10.0
Delaware	12 (5.3)	174 (1.6)	6.9	14 (6.1)	293 (2.8)	4.8	29 (12.7)	722 (6.8)	4.0	43 (18.9)	1,015 (9.6)	4.2
District of Columbia	—^¶^ (0.5)	181 (2.0)	1.1	8 (1.9)	311 (3.4)	2.6	60 (14.1)	619 (6.7)	9.7	68 (15.9)	930 (10.1)	7.3
Florida	180 (5.6)	3,804 (1.7)	4.7	318 (9.9)	6,457 (2.9)	4.9	673 (20.9)	16,244 (7.3)	4.1	991 (30.8)	22,701 (10.2)	4.4
Georgia	67 (3.7)	2,539 (2.0)	2.6	125 (6.8)	4,340 (3.4)	2.9	416 (22.8)	10,169 (8.1)	4.1	541 (29.6)	14,509 (11.5)	3.7
Hawaii	29 (6.8)	270 (1.6)	10.7	48 (11.3)	459 (2.7)	10.5	85 (20.0)	1,285 (7.6)	6.6	133 (31.3)	1,744 (10.3)	7.6
Idaho	27 (8.7)	265 (1.2)	10.2	50 (16.0)	463 (2.2)	10.8	71 (22.8)	1,470 (6.9)	4.8	121 (38.8)	1,933 (9.0)	6.3
Illinois	156 (3.5)	2,401 (1.7)	6.5	330 (7.4)	4,292 (3.0)	7.7	811 (18.2)	11,153 (7.7)	7.3	1,141 (25.6)	15,445 (10.7)	7.4
Indiana	43 (5.2)	1,307 (1.6)	3.3	73 (8.8)	2,254 (2.8)	3.2	199 (24.0)	6,097 (7.5)	3.3	272 (32.8)	8,351 (10.2)	3.3
Iowa	31 (4.5)	530 (1.4)	5.8	70 (10.2)	993 (2.6)	7.0	140 (20.3)	2,752 (7.3)	5.1	210 (30.5)	3,745 (9.9)	5.6
Kansas	12 (3.0)	511 (1.4)	2.3	29 (7.2)	922 (2.5)	3.1	70 (17.5)	2,518 (6.9)	2.8	99 (24.7)	3,440 (9.5)	2.9
Kentucky	21 (4.0)	878 (1.6)	2.4	54 (10.2)	1,607 (3.0)	3.4	105 (19.8)	4,502 (8.3)	2.3	159 (30.0)	6,109 (11.3)	2.6
Louisiana	29 (5.4)	1,293 (2.2)	2.2	50 (9.3)	2,167 (3.6)	2.3	119 (22.2)	5,576 (9.4)	2.1	169 (31.6)	7,743 (13.0)	2.2
Maine	7 (3.4)	140 (1.1)	5.0	15 (7.3)	266 (2.2)	5.6	36 (17.6)	788 (6.4)	4.6	51 (24.9)	1,054 (8.6)	4.8
Maryland	85 (3.9)	1,308 (1.8)	6.5	159 (7.3)	2,189 (3.1)	7.3	342 (15.6)	5,042 (7.1)	6.8	501 (22.9)	7,231 (10.2)	6.9
Massachusetts	85 (2.4)	921 (1.3)	9.2	199 (5.6)	1,682 (2.4)	11.8	556 (15.7)	4,490 (6.5)	12.4	755 (21.4)	6,172 (8.9)	12.2
Michigan	76 (4.3)	1,686 (1.5)	4.5	176 (9.9)	2,973 (2.7)	5.9	351 (19.8)	8,066 (7.3)	4.4	527 (29.8)	11,039 (10.0)	4.8
Minnesota	70 (5.0)	890 (1.3)	7.9	125 (9.0)	1,551 (2.3)	8.1	246 (17.6)	4,453 (6.6)	5.5	371 (26.6)	6,004 (8.9)	6.2
Mississippi	19 (7.8)	888 (2.4)	2.1	32 (13.2)	1,536 (4.2)	2.1	45 (18.5)	3,733 (10.1)	1.2	77 (31.7)	5,269 (14.2)	1.5
Missouri	33 (3.4)	1,184 (1.6)	2.8	85 (8.8)	2,072 (2.8)	4.1	216 (22.5)	5,777 (7.9)	3.7	301 (31.3)	7,849 (10.7)	3.8
Montana	—^¶^ (3.0)	123 (1.1)	3.3	14 (10.5)	257 (2.2)	5.4	19 (14.3)	790 (6.9)	2.4	33 (24.8)	1,047 (9.1)	3.2
Nebraska	9 (2.6)	370 (1.5)	2.4	27 (7.8)	665 (2.6)	4.1	111 (32.0)	1,999 (7.8)	5.6	138 (39.8)	2,664 (10.5)	5.2
Nevada	41 (7.3)	532 (1.5)	7.7	64 (11.4)	962 (2.7)	6.7	102 (18.1)	2,654 (7.4)	3.8	166 (29.5)	3,616 (10.1)	4.6
New Hampshire	11 (2.5)	152 (1.3)	7.2	30 (6.9)	276 (2.3)	10.9	68 (15.6)	719 (6.0)	9.5	98 (22.4)	995 (8.3)	9.8
New Jersey	136 (3.2)	1,518 (1.5)	9.0	258 (6.1)	2,655 (2.6)	9.7	660 (15.6)	6,963 (6.9)	9.5	918 (21.8)	9,618 (9.5)	9.5
New Mexico	—^¶^ (2.2)	327 (1.4)	1.2	12 (6.5)	580 (2.5)	2.1	49 (26.5)	1,674 (7.3)	2.9	61 (33.0)	2,254 (9.8)	2.7
New York	270 (3.5)	3,266 (1.4)	8.3	494 (6.4)	5,799 (2.6)	8.5	1,206 (15.7)	14,482 (6.4)	8.3	1,700 (22.1)	20,281 (9.0)	8.4
North Carolina	77 (4.2)	2,095 (1.8)	3.7	157 (8.5)	3,581 (3.0)	4.4	364 (19.7)	8,759 (7.4)	4.2	521 (28.2)	12,340 (10.4)	4.2
North Dakota	11 (8.3)	128 (1.2)	8.6	18 (13.6)	262 (2.5)	6.9	32 (24.2)	756 (7.1)	4.2	50 (37.9)	1,018 (9.6)	4.9
Ohio	66 (3.6)	2,262 (1.7)	2.9	139 (7.6)	3,896 (2.9)	3.6	300 (16.3)	9,949 (7.4)	3.0	439 (23.9)	13,845 (10.2)	3.2
Oklahoma	18 (4.6)	823 (1.7)	2.2	38 (9.7)	1,478 (3.0)	2.6	93 (23.7)	4,192 (8.4)	2.2	131 (33.4)	5,670 (11.4)	2.3
Oregon	51 (6.8)	437 (1.0)	11.7	81 (10.7)	813 (1.9)	10.0	119 (15.8)	2,491 (5.9)	4.8	200 (26.5)	3,304 (7.8)	6.1
Pennsylvania	85 (3.4)	2,124 (1.6)	4.0	161 (6.4)	3,688 (2.7)	4.4	446 (17.6)	9,227 (6.8)	4.8	607 (24.0)	12,915 (9.5)	4.7
Puerto Rico	9 (10.7)	459 (2.1)	2.0	15 (17.9)	863 (4.0)	1.7	26 (31.0)	2,235 (10.4)	1.2	41 (48.8)	3,098 (14.5)	1.3
Rhode Island	23 (7.6)	143 (1.4)	16.1	29 (9.6)	276 (2.6)	10.5	48 (15.9)	667 (6.3)	7.2	77 (25.6)	943 (9.0)	8.2
South Carolina	25 (3.6)	1,083 (1.9)	2.3	61 (8.9)	1,855 (3.3)	3.3	143 (20.8)	4,571 (8.1)	3.1	204 (29.6)	6,426 (11.3)	3.2
South Dakota	8 (6.3)	141 (1.2)	5.7	17 (13.3)	254 (2.1)	6.7	22 (17.2)	868 (7.3)	2.5	39 (30.5)	1,122 (9.4)	3.5
Tennessee	46 (5.8)	1,441 (1.8)	3.2	84 (10.6)	2,531 (3.1)	3.3	153 (19.3)	6,380 (7.9)	2.4	237 (29.9)	8,911 (11.0)	2.7
Texas	335 (5.4)	6,182 (1.6)	5.4	560 (9.1)	10,983 (2.9)	5.1	1,375 (22.4)	29,816 (7.9)	4.6	1,935 (31.5)	40,799 (10.8)	4.7
Utah	52 (4.8)	600 (1.3)	8.7	101 (9.3)	1,053 (2.2)	9.6	260 (24.0)	3,392 (7.2)	7.7	361 (33.3)	4,445 (9.4)	8.1
Vermont	—^¶^ (2.5)	66 (1.2)	4.5	12 (10.1)	116 (2.1)	10.3	20 (16.8)	346 (6.4)	5.8	32 (26.9)	462 (8.5)	6.9
Virginia	100 (4.3)	1,613 (1.6)	6.2	178 (7.6)	2,643 (2.6)	6.7	366 (15.7)	6,758 (6.8)	5.4	544 (23.3)	9,401 (9.4)	5.8
Washington	49 (2.8)	1,020 (1.2)	4.8	116 (6.7)	1,854 (2.2)	6.3	284 (16.4)	5,293 (6.1)	5.4	400 (23.1)	7,147 (8.3)	5.6
West Virginia	—^¶^ (2.5)	311 (1.7)	1.0	9 (7.5)	566 (3.1)	1.6	27 (22.5)	1,592 (8.7)	1.7	36 (30.0)	2,158 (11.8)	1.7
Wisconsin	32 (3.5)	924 (1.4)	3.5	82 (8.9)	1,664 (2.6)	4.9	206 (22.5)	4,684 (7.3)	4.4	288 (31.4)	6,348 (9.9)	4.5
Wyoming	—^¶^ (2.0)	92 (1.4)	2.2	5 (5.1)	157 (2.4)	3.2	29 (29.6)	489 (7.5)	5.9	34 (34.7)	646 (9.8)	5.3
**Total**	**2,968 (4.0)**	**59,790 (1.6)**	**5.0**	**5,805 (7.8)**	**104,894 (2.8)**	**5.5**	**13,706 (18.4)**	**277,981 (7.3)**	**4.9**	**19,511 (26.1)**	**382,875 (10.0)**	**5.1**

**Abbreviations:** ART = assisted reproductive technology; PTB = preterm birth; VPTB = very preterm birth.

* In cases of missing patient’s residence data (0.3%), the patient’s residence was assigned as the location where the ART procedure was performed.

^†^ ART totals include infants conceived from ART procedures performed in 2017 and born in 2018 and infants conceived from ART procedures performed in 2018 and born in 2018. Total ART births exclude births to non-U.S. residents and include only infants with gestational age data available.

^§^ U.S. births exclude births to non-U.S. residents **(Source:** National Center for Health Statistics, Vital statistics data available. Natality public use file and CD-ROM. Hyattsville, MD, National Center for Health Statistics).

^¶^ To protect confidentiality, cells with values of 1–4 for ART infants and cells with values of 0–9 for all infants are suppressed. Also suppressed are data that can be used to derive suppressed cell values. These values are included in the totals.

Among singletons only, the percentage of infants who had low birthweight was 8.3% among ART-conceived infants and 6.6% among all infants. In addition, among singletons, the percentage of infants who were born preterm was 14.9% among ART-conceived infants and 8.3% among all infants, and the percentage of SGA infants was 7.3% among ART-conceived infants and 9.4% for all infants (Table 7).

**TABLE 7:** Percentage of low birthweight, preterm, and small for gestational age infants among singleton infants born with assisted reproductive technology, by female patient’s area of residence at time of treatment — United States and Puerto Rico, 2018

Patient’s area of residence*	Low birthweight (<2,500 g)		Preterm (<37 wks)		Small for gestational age (22–44 wks)	
	ART infants^†^ (%)	All infants^§^ (%)	ART infants^†^ (%)	All infants^§^ (%)	ART infants^†^ (%)	All infants^§^ (%)
Alabama	8.2	8.7	15.2	10.4	6.9	11.6
Alaska	—^¶^	4.4	10.9	7.5	—^¶^	5.7
Arizona	10.3	5.9	17.2	7.7	6.7	8.7
Arkansas	8.7	7.6	13.5	9.8	8.1	9.9
California	7.5	5.5	12.8	7.2	7.8	8.7
Colorado	9.0	7.6	15.1	7.5	9.9	12.8
Connecticut	8.1	5.9	12.8	7.6	7.4	8.4
Delaware	9.6	7.6	16.4	8.2	6.8	10.2
District of Columbia	7.2	8.4	11.6	8.4	8.4	11.5
Florida	8.0	7.1	17.4	8.6	6.7	9.9
Georgia	8.2	8.2	15.8	9.5	6.8	11.3
Hawaii	14.3	6.9	18.2	8.7	12.6	10.4
Idaho	9.5	5.6	18.7	7.2	6.0	8.8
Illinois	8.6	6.8	16.2	8.8	7.3	9.0
Indiana	8.4	6.4	16.4	8.4	4.6	8.7
Iowa	8.2	5.3	18.1	8.0	5.2	6.8
Kansas	6.5	5.8	13.5	7.7	5.8	7.8
Kentucky	6.7	7.1	13.1	9.4	6.7	8.7
Louisiana	8.6	8.9	17.4	10.9	5.0	10.9
Maine	5.4	5.7	11.3	7.0	5.4	8.2
Maryland	9.0	7.1	15.6	8.3	6.8	9.6
Massachusetts	8.4	5.8	13.1	7.0	8.3	8.9
Michigan	9.9	6.7	14.6	8.1	7.9	9.5
Minnesota	8.0	5.3	13.2	7.2	6.7	7.4
Mississippi	13.7	10.1	19.0	12.1	5.3	12.0
Missouri	8.2	6.9	16.3	8.6	7.4	9.0
Montana	7.3	6.0	12.7	7.5	9.1	9.3
Nebraska	8.9	5.8	20.5	8.4	8.1	7.4
Nevada	11.2	7.1	16.4	8.6	8.7	10.8
New Hampshire	5.9	5.2	11.0	6.6	6.5	7.8
New Jersey	7.7	6.1	14.2	7.7	7.4	9.5
New Mexico	15.0	7.7	21.1	8.3	13.7	12.2
New York	8.0	6.3	13.2	7.2	8.1	10.0
North Carolina	7.9	7.5	14.8	8.5	7.5	10.2
North Dakota	—^¶^	4.9	12.4	7.6	—^¶^	6.3
Ohio	8.1	6.8	13.8	8.4	7.2	9.3
Oklahoma	7.1	6.6	14.4	9.4	5.2	8.3
Oregon	5.9	5.1	12.7	6.3	5.7	8.1
Pennsylvania	8.1	6.7	15.3	7.9	5.7	9.6
Puerto Rico	21.7	9.2	32.6	13.4	13.0	13.5
Rhode Island	6.5	5.8	12.5	7.1	9.1	8.7
South Carolina	8.8	7.9	16.7	9.5	6.2	10.1
South Dakota	10.2	5.0	18.9	7.5	—^¶^	7.6
Tennessee	9.9	7.5	17.7	9.2	5.7	10.0
Texas	9.6	6.8	18.1	9.0	6.4	9.2
Utah	12.0	5.3	17.5	7.4	9.8	8.1
Vermont	8.0	5.6	17.8	7.0	9.3	8.0
Virginia	7.5	6.5	14.7	7.7	6.5	9.5
Washington	8.2	5.3	13.6	6.9	6.4	7.8
West Virginia	9.6	7.6	18.6	9.9	7.2	9.7
Wisconsin	6.7	5.9	15.0	7.9	6.1	8.0
Wyoming	12.3	7.5	18.2	8.0	9.2	13.0
**Total**	**8.3**	**6.6**	**14.9**	**8.3**	**7.3**	**9.4**

**Abbreviation:** ART = assisted reproductive technology.

* In cases of missing patient’s residence data (0.3%), the patient’s residence was assigned as the location where the ART procedure was performed.

^†^ ART totals include infants conceived from ART procedures performed in 2017 and born in 2018 and infants conceived from ART procedures performed in 2018 and born in 2018. Total ART births exclude births to non- U.S. residents and include only infants with gestational age data available.

^§^ U.S. births exclude births to non-U.S. residents (**Source:** National Center for Health Statistics, Vital statistics data available. Natality public use file and CD-ROM. Hyattsville, MD, National Center for Health Statistics).

^¶^ To protect confidentiality, cells with values of 1–4 for ART infants and cells with values of 0–9 for all infants are suppressed. Also suppressed are data that can be used to derive suppressed cell values. These values are included in the totals.

## Discussion

### Overview

The use of ART has increased substantially in the United States since the beginning of ART surveillance. In 1996 (the first full year for which ART data were reported to CDC), 20,597 infants were born from 64,036 ART procedures performed by 302 reporting clinics (*2*). Since then, the number of clinics performing ART services has increased considerably, the number of reported procedures has tripled, and the number of infants born from ART procedures has almost quadrupled. Multiple improvements can be observed in embryo-transfer practices and ART outcomes by comparing data for years 2017 and 2018 (*21*). The rate of SET procedures increased from 67.3% to 74.1% among women aged <35 years (a 10.1% increase), from 65.0% to 72.8% among women aged 35–37 years (a 12.0% increase), and from 60.0% to 66.4% among women aged >37 years (a 10.7% increase). Relatedly, from 2017 to 2018 the percentage of singleton births increased from 73.6% to 78.6% (a 6.8% increase), the percentage of twin births decreased from 25.5% to 20.7% (a 18.8% decrease), and the percentage of triplets and higher-order multiples decreased from 0.9% to 0.6% (a 33.3% decrease). The percentage of low birthweight among ART-conceived infants decreased from 20.2% to 18.3% (a 9.4% decrease), and preterm birth rates decreased from 27.8% to 26.1% (a 6.1% decrease). The contribution of ART-conceived twins to all twins born in the United States decreased from 14.7% to 12.5% (a 15.0% decrease). The contribution of ART-conceived infants to all triplets and higher-order infants decreased from 17.3% to 13.3% (a 23.1% decrease).

Despite these improvements, ART still disproportionally contributes to multiple births and poor birth outcomes (low birthweight and preterm birth). Because births resulting from ART are more likely to be multiple births than are births in the general population, and because multiple births are associated with higher rates of prematurity than singleton births, their contribution to poor birth outcomes continues to be noteworthy. In 2018, the multiple birth rate was nearly 6.5 times higher among ART-conceived infants compared with all infants (21.4% versus 3.3%). Although infants conceived with ART accounted for approximately 2.0% of total births in the United States in 2018, the proportion of multiple births attributable to ART was 12.5%. The percentage of infants with low birthweight or born preterm was 2.2 and 2.6 times higher among ART-conceived infants (18.3% and 26.1%, respectively) than among all infants (8.3% and 10.0%, respectively). Nationally, even among singletons, the rate of preterm birth among ART-conceived infants was 1.8 times the preterm birth rate among all infants (14.9% and 8.3%, respectively).

### Variations in ART Use by States and Territories

The rate of ART use, as measured by number of procedures performed per 1 million women aged 15–44 years, increased from 3,040 in reporting year 2017 to 3,135 in reporting year 2018. ART use varied across areas. Residents of 14 states (California, Connecticut, Delaware, Hawaii, Illinois, Maryland, Massachusetts, New Hampshire, New Jersey, New York, Rhode Island, Utah, Vermont, and Virginia) and the District of Columbia had higher rates of ART use than the national rate. Residents of California, Illinois, Massachusetts, New Jersey, New York, and Texas accounted for almost half (46.1%) of all infants conceived with ART. The large number of ART procedures performed in these six states is a result of the size of the general population (California and Texas), high rates of ART use per capita (Illinois, Massachusetts, and New Jersey), or both (New York).

The contribution of ART to all infants born varied substantially by state. State-level differences might be explained in part by variations in insurance mandates for infertility treatments and disparities in access to fertility services (*22*–*24*). As of 2021, a total of 19 states (Arkansas, California, Colorado, Connecticut, Delaware, Hawaii, Illinois, Louisiana, Maryland, Massachusetts, Montana, New Hampshire, New Jersey, New York, Ohio, Rhode Island, Texas, Utah, and West Virginia) had passed fertility insurance coverage laws mandating that private insurers provide coverage for certain fertility treatments, although not all mandates require coverage for ART (*25*,*26*). Of four states that provide comprehensive coverage, two include at least four oocyte retrievals (Illinois and New Jersey), one does not limit the number of treatment cycles (Massachusetts), and one has a $100,000 cap on treatment (Rhode Island). These four states had rates of ART use that were ≥50% higher than the national rate.

Other possible contributors to differences in ART use across states might include state variations in demographic characteristics such as race, ethnicity, and income levels. Use of fertility services varies by race and ethnicity. One study that analyzed 2014 NASS data indicated that ART use was highest among Asians or Pacific Islanders, followed by non-Hispanic White women, whereas non-Hispanic Black, Hispanic, and non-Hispanic American Indian or Alaska Native women had substantially lower levels of ART use (*27*). A study that linked NASS data to state vital statistics registries data in Florida, Massachusetts, and Michigan also found disparities by maternal race and ethnicity in ART use (*24*). Because many insurance plans in the United States do not provide coverage for ART treatment, costs often are the responsibility of patients (*22*). Even in states with an insurance mandate, ART use rates for non-Hispanic Black and Hispanic women were substantially lower than the rate for non-Hispanic White women (*27*). Thus, differences in the use rate might be due to factors beyond costs (*27*). Disparities might be due to cultural and social barriers, such as stigma, which might delay or prevent certain racial and ethnic groups from seeking infertility care (*28*).

Limitations of current data collection systems in terms of data quality and completeness might limit meaningful research in racial and ethnic disparities in ART use and outcomes (*27*). As of January 2016, all states had adopted the 2003 revision of the birth certificate that includes information on whether the pregnancy resulted from the use of infertility treatment; 47 states and the District of Columbia differentiate between the use of ART and non-ART treatments. This additional information on ART use in birth certificates could facilitate more comprehensive research on racial and ethnic differences in ART use and outcomes among infants born in the United States. The scope of such research would be greater if data from NASS, which include detailed information on ART, were to be linked to data from state vital statistics registries.

### Single-Embryo Transfer Rates

Recommendations issued by the American Society for Reproductive Medicine and SART to limit the number of embryos to transfer have been revised multiple times to reduce higher-order multiple deliveries (*29*–*32*). The most recent guidance was intended to reduce all multiple births, including twins (*3*). This guidance adopted a broader approach and recommended SET for patients of any age transferring a euploid embryo, selected with the assistance of preimplantation genetic screening, and for patients aged <38 years with any one of the following criteria: 1) availability of quality embryos for cryopreservation, 2) a history of live birth after an IVF procedure, 3) availability of vitrified blastocyst stage embryos, or 4) undergoing first frozen-embryo transfer (*3*). Increasing SET rates among all age groups reflect these changes in clinical practice. However, variations in the percentages of SET procedures across states and territories suggest that SET has not been implemented equally in all areas.

### ART Multiple Births

Singleton births have lower risks than multiple births for adverse outcomes, such as prematurity, low birthweight, developmental disability, and death (*10*,*33*,*34*). To optimize healthy birth outcomes, the transfer of fewer embryos should be encouraged when clinically appropriate, taking into consideration the patient’s age and prognosis (*3*,*35*). The percentage of multiple births among ART-conceived infants in the United States has decreased from 53.1% in 2000 (when national multiple birth rates were first reported) to 21.4% in 2018 (*36*). A substantial decrease was noted for both the percentage of ART-conceived triplets and higher-order multiples (from 8.9% in 2000 to 0.6% in 2018) and the percentage of ART-conceived twins (from 44.2% in 2000 to 20.7% in 2018). Areas with the highest SET rates (Delaware, the District of Columbia, and Massachusetts) also had the lowest rates of ART-conceived multiple births.

Transferring two embryos is associated with a slight increase in overall birth rate but a much greater increase in the twin birth rate compared with transferring a single embryo (*37*,*38*). However, transferring two embryos sequentially (single-embryo transfer over two sequential procedures, if the first procedure did not result in a live birth) has similar cumulative live-birth rates and substantially lower twin delivery rates than transferring two embryos in a single procedure and might be a cost-effective approach; estimated costs include ART treatment and pregnancy- and infant-associated medical costs (*37*,*38*). Evidence from other countries suggests that access to coverage for ART, availability of cryopreservation services, and economic and social factors regarding the number of embryos transferred per cycle can encourage SET procedures and reduce multiple births (*39*). In 2013, the mean health care costs to patients and insurers were estimated at $26,922 for ART-conceived singleton deliveries, $115,238 for ART-conceived twin deliveries, and $434,668 for ART-conceived triplets and higher-order infants (*40*).

The desire for twins among couples experiencing infertility and their perception that the benefits of a multiple-gestation pregnancy (compared with no pregnancy) outweigh the risks (*41*–*43*) might partially explain why twin rates remain high. Therefore, understanding the perspective of couples undergoing infertility treatments regarding multiple-gestation pregnancies and multiple births is important. Patient education focusing on maternal and perinatal morbidity and mortality and the economic costs of twin gestations has been effective in reducing the preference for twins among patients undergoing ART (*44*–*46*).

### Low Birthweight, Preterm Birth, and Small for Gestational Age Among ART Infants

In the United States, although rates of ART-conceived preterm and low birthweight infants have been declining steadily, the percentage of infants born with low birthweight and born preterm remained higher among ART-conceived infants (18.3% and 26.1%, respectively) than among all infants (8.3% and 10.0%, respectively). In addition, preterm and low birthweight rates varied more widely across areas among ART-conceived infants than among all infants.

Fertility treatments, both ART and non-ART, contribute substantially to preterm birth, which is a leading cause of infant morbidity and mortality (*33*,*47*–*49*). The health risks associated with preterm birth have contributed to increased health care costs. In 2016, the societal economic cost associated with one infant born preterm in the United States was estimated at $64,815 (*33*,*50*), which translates into approximately $1.3 billion for 19,511 ART-conceived infants born preterm in 2018.

Although ART-conceived singletons had higher rates of preterm birth (14.9%) than singletons among the general population (8.3%), SGA rates were lower among the former. Studies have indicated that SGA risks among ART-conceived infants might be lower than among non-ART infants and also might vary by type of ART performed (*51*–*53*). ART singletons from fresh-embryo transfers had higher risks for SGA than non-ART singletons, whereas ART singletons from frozen-embryo transfers had lower risks for SGA than non-ART singletons (*52*). In a more recent study, SGA rates were lower among ART singletons from frozen-embryo transfers than singletons from both fresh-embryo transfers and infants conceived naturally; genetic factors as well as the freeze-thaw process might explain these differences (*53*). More research is needed to better understand the risks for SGA among ART-conceived infants and how these risks might vary by the type of ART cycle performed.

Use of ART only partially explains the overall prevalence of multiple births in the United States. Other factors influencing multiple births include advanced maternal age at conception and the use of non-ART fertility treatments (*33*,*54*–*56*). The risk for multiple gestations associated with non-ART fertility treatments (i.e., controlled ovarian stimulation and ovulation induction coupled with timed intercourse or intrauterine insemination) is less well documented than that associated with ART procedures; fertility clinics are only required to report data on ART use to NASS. However, research suggests that non-ART fertility treatments contribute to a larger percentage of multiple births than ART. In 2015, approximately 17% of multiple births in the United States were attributable to IVF fertility treatments, whereas 29% were attributable to non-IVF fertility treatments (*57*,*58*).

Additional efforts are needed to monitor the use of non-ART fertility treatments and their role in multiple births, particularly because the ability to control the occurrence of a multiple birth is more challenging when using non-ART fertility treatments (*54*). CDC is monitoring the prevalence of ART and non-ART fertility treatment use and resultant outcomes among women who had live births in states participating in the Pregnancy Risk Assessment Monitoring System (*59*,*60*).

## Limitations

The findings in this report are subject to at least seven limitations. First, the procedure-specific use rates might be higher than the actual per-patient use rates. ART surveillance data were reported for each ART procedure performed rather than for each patient who used ART, and patients can undergo more than one procedure during the reporting period. In addition, the procedure-specific use rates are standardized by the size of the female population aged 15–44 years and do not account for certain ART users who are aged >44 years. Second, when comparisons are made between ART-conceived births and all births, all births also include ART-related births. Third, preterm birth, low birthweight, and SGA could be associated with factors contributing to underlying infertility or other maternal or paternal factors and not necessarily ART procedures. Fourth, approximately 9% of fertility clinics that performed ART in 2018 did not report their data to CDC. Although these clinics might have had results differing from reporting clinics, typically they are smaller and represent approximately 2% of all ART cycles performed in the United States (*1*). Fifth, gestational age is computed for ART infants conceived with frozen embryos by subtracting 17 days (to allow for an average of 3 days in embryo culture) from the date of transfer. However, many frozen-embryo transfers use blastocyst embryos (approximately 5 days of embryo culture), which might slightly underestimate gestational age. Sixth, SET rates cannot be compared with elective single embryo transfer (eSET) rates from previous reports (2016 and prior) because of differences in definition between eSET (a procedure in which one embryo is selected for transfer from a larger number of available embryos, and the remaining embryos are cryopreserved) and SET (a procedure in which one embryo is transferred regardless of the number of available embryos). In addition, in previous reports, eSET rates were reported only for procedures in which patients were using their own fresh eggs. Finally, the number of ART procedures reported for 2018 included all procedures in which banking was not performed, including procedures with frozen eggs that were thawed. Therefore, comparisons with previous years (2015 and prior) in which procedures using thawed eggs were excluded from analyses should be made with caution.

## Conclusion

Since 1995, the number of ART procedures performed and the number of infants born as a result have more than tripled in the United States. With this increasing use, ART-conceived infants represented approximately 2% of infants born in 2018 in the United States and contributed to approximately 4% and 5% of all low birthweight and preterm births, respectively. Although the percentage of all multiple births including twins, triplets, and higher-order multiples has decreased among ART-conceived infants since 2000, the percentage of twins remains high (21%). Because multiple births have higher rates of preterm birth than singleton births, ART has a disproportionate impact on poor birth outcomes. This report provides information that allows state health departments to monitor and inform patients of the extent of ART use and ART-related adverse perinatal outcomes in their regions and take action to initiate programs and policies that improve access to ART treatments and reduce the adverse effects of ART multiple births. A state-specific website that presents key ART success rates and other statistics is available at https://www.cdc.gov/art/state-specific-surveillance/index.html.

## References

[1] CDC. 2018 assisted reproductive technology fertility clinic success rates report. Atlanta, GA: US Department of Health and Human Services, CDC; 2020. https://www.cdc.gov/art/reports/2018/fertility-clinic.html

[2] CDC. 1995 assisted reproductive technology fertility clinic success rates report. Atlanta, GA: US Department of Health and Human Services, CDC; 1997.

[3] Penzias A, Bendikson K, Butts S, ; Practice Committee of the American Society for Reproductive Medicine. Guidance on the limits to the number of embryos to transfer: a committee opinion. Fertil Steril 2017;107:901–3. 10.1016/j.fertnstert.2017.02.107 28292618

[4] Di Tommaso M, Sisti G, Colombi I, Influence of assisted reproductive technologies on maternal and neonatal outcomes in early preterm deliveries. J Gynecol Obstet Hum Reprod 2019;48:845–8. 10.1016/j.jogoh.2019.03.008 30898633

[5] Le Ray C, Pelage L, Seco A, ; Epimoms Study Group. Risk of severe maternal morbidity associated with in vitro fertilisation: a population-based study. BJOG 2019;126:1033–41. 10.1111/1471-0528.15668 30801948

[6] Santana DS, Surita FG, Cecatti JG. Multiple pregnancy: epidemiology and association with maternal and perinatal morbidity. Rev Bras Ginecol Obstet 2018;40:554–62. 10.1055/s-0038-1668117 30231294 PMC10316907

[7] MacKay AP, Berg CJ, King JC, Duran C, Chang J. Pregnancy-related mortality among women with multifetal pregnancies. Obstet Gynecol 2006;107:563–8. 10.1097/01.AOG.0000200045.91015.c6 16507925

[8] Fountain C, Zhang Y, Kissin DM, Association between assisted reproductive technology conception and autism in California, 1997–2007. Am J Public Health 2015;105:963–71. 10.2105/AJPH.2014.302383 25790396 PMC4386543

[9] Boulet SL, Kirby RS, Reefhuis J, ; States Monitoring Assisted Reproductive Technology (SMART) Collaborative. Assisted reproductive technology and birth defects among liveborn infants in Florida, Massachusetts, and Michigan, 2000–2010. JAMA Pediatr 2016;170:e154934. 10.1001/jamapediatrics.2015.4934 27043648 PMC4899282

[10] Boulet SL, Schieve LA, Nannini A, Perinatal outcomes of twin births conceived using assisted reproduction technology: a population-based study. Hum Reprod 2008;23:1941–8. 10.1093/humrep/den169 18487216

[11] Dawson AL, Tinker SC, Jamieson DJ, ; National Birth Defects Prevention Study. Twinning and major birth defects, National Birth Defects Prevention Study, 1997–2007. J Epidemiol Community Health 2016;70:1114–21. 10.1136/jech-2015-206302 27325867 PMC5299593

[12] Qin JB, Sheng XQ, Wu D, Worldwide prevalence of adverse pregnancy outcomes among singleton pregnancies after in vitro fertilization/intracytoplasmic sperm injection: a systematic review and meta-analysis. Arch Gynecol Obstet 2017;295:285–301. 10.1007/s00404-016-4250-3 27896474

[13] Martin AS, Chang J, Zhang Y, ; States Monitoring Assisted Reproductive Technology (SMART) Collaborative. Perinatal outcomes among singletons after assisted reproductive technology with single-embryo or double-embryo transfer versus no assisted reproductive technology. Fertil Steril 2017;107:954–60. 10.1016/j.fertnstert.2017.01.024 28292615 PMC11350526

[14] Farr SL, Schieve LA, Jamieson DJ. Pregnancy loss among pregnancies conceived through assisted reproductive technology, United States, 1999–2002. Am J Epidemiol 2007;165:1380–8. 10.1093/aje/kwm035 17351291

[15] Hipp H, Crawford S, Kawwass JF, Chang J, Kissin DM, Jamieson DJ. First trimester pregnancy loss after fresh and frozen in vitro fertilization cycles. Fertil Steril 2016;105:722–8 10.1016/j.fertnstert.2015.11.012 26627121 PMC11057006

[16] National Center for Health Statistics. Vital statistics data available. Natality public use file and CD-ROM. Hyattsville, MD: National Center for Health Statistics.

[17] Martin JA, Hamilton BE, Osterman MJK, Driscoll AK. Births: Final Data for 2018. Natl Vital Stat Rep 2019;68:1–47. 32501202

[18] US Census Bureau. Annual estimates of the resident population for selected age groups by sex for the United States, states, counties, and Puerto Rico Commonwealth and municipios: April 1, 2010 to July 1, 2018. Washington, DC: US Census Bureau, Population Division; 2018. https://data.census.gov/cedsci/table?q=United%20States&g=0100000US&tid=ACSST1Y2018.S0101&vintage=2018

[19] Martin JA, Osterman MJ, Kirmeyer SE, Gregory EC. Measuring gestational age in vital statistics data: transitioning to the obstetric estimate. Natl Vital Stat Rep 2015;64:1–20. 26047089

[20] Talge NM, Mudd LM, Sikorskii A, Basso O. United States birth weight reference corrected for implausible gestational age estimates. Pediatrics 2014;133:844–53. 10.1542/peds.2013-3285 24777216

[21] Sunderam S, Kissin DM, Zhang Y, Assisted reproductive technology surveillance—United States, 2017. MMWR Surveill Summ 2020;69:1–20. 10.15585/mmwr.ss6909a1 33332294 PMC7755269

[22] Henne MB, Bundorf MK. Insurance mandates and trends in infertility treatments. Fertil Steril 2008;89:66–73. 10.1016/j.fertnstert.2007.01.167 17482603

[23] Hamilton BH, McManus B. The effects of insurance mandates on choices and outcomes in infertility treatment markets. Health Econ 2012;21:994–1016. 10.1002/hec.1776 21905150

[24] Boulet SL, Crawford S, Zhang Y, ; States Monitoring ART Collaborative. Embryo transfer practices and perinatal outcomes by insurance mandate status. Fertil Steril 2015;104:403–9.e1. 10.1016/j.fertnstert.2015.05.015 26051096 PMC4522367

[25] Zagadailov P, Seifer DB, Shan H, Zarek SM, Hsu AL. Do state insurance mandates alter ICSI utilization? Reprod Biol Endocrinol 2020;18:33. 10.1186/s12958-020-00589-w 32334609 PMC7183130

[26] RESOLVE. Infertility coverage in your state. McLean, VA: RESOLVE. https://resolve.org/what-are-my-options/insurance-coverage/infertility-coverage-state/

[27] Dieke AC, Zhang Y, Kissin DM, Barfield WD, Boulet SL. Disparities in assisted reproductive technology utilization by race and ethnicity, United States, 2014: a commentary. J Womens Health (Larchmt) 2017;26:605–8. 10.1089/jwh.2017.6467 28586255 PMC5548290

[28] Quinn M, Fujimoto V. Racial and ethnic disparities in assisted reproductive technology access and outcomes. Fertil Steril 2016;105:1119–23. 10.1016/j.fertnstert.2016.03.007 27054308

[29] Practice Committee of Society for Assisted Reproductive Technology; Practice Committee of American Society for Reproductive Medicine. Guidelines on number of embryos transferred. Fertil Steril 2008;90(Suppl):S163–4. 10.1016/j.fertnstert.2008.08.053 19007617

[30] Practice Committee of the American Society for Reproductive Medicine; Practice Committee of the Society for Assisted Reproductive Technology. Guidelines on number of embryos transferred. Fertil Steril 2009;92:1518–9. 10.1016/j.fertnstert.2009.08.059 19836732

[31] Practice Committee of the Society for Assisted Reproductive Technology; Practice Committee of the American Society for Reproductive Medicine. Guidelines on number of embryos transferred. Fertil Steril 2006;86(Suppl 1):S51–2. 10.1016/j.fertnstert.2006.07.1473 17055845

[32] Practice Committee of the American Society for Reproductive Medicine; Practice Committee of the Society for Assisted Reproductive Technology. Criteria for number of embryos to transfer: a committee opinion. Fertil Steril 2013;99:44–6. 10.1016/j.fertnstert.2012.09.038 23095140

[33] Institute of Medicine, Board on Health Sciences Policy, Committee on Understanding Premature Birth and Assuring Healthy Outcomes. Premature birth: causes, consequences, and prevention. Behrman RE, Butler AS, eds. Washington, DC: National Academies Press; 2007.

[34] Practice Committee of American Society for Reproductive Medicine. Multiple gestation associated with infertility therapy: an American Society for Reproductive Medicine Practice Committee opinion. Fertil Steril 2012;97:825–34. 10.1016/j.fertnstert.2011.11.048 22192352

[35] Kissin DM, Kulkarni AD, Kushnir VA, Jamieson DJ; National ART Surveillance System Group. Number of embryos transferred after in vitro fertilization and good perinatal outcome. Obstet Gynecol 2014;123:239–47. 10.1097/AOG.0000000000000106 24402601 PMC4607031

[36] Wright VC, Schieve LA, Reynolds MA, Jeng G. Assisted reproductive technology surveillance—United States, 2000. MMWR Surveill Summ 2003;52:1–16. 14532867

[37] Luke B, Brown MB, Wantman E, Application of a validated prediction model for in vitro fertilization: comparison of live birth rates and multiple birth rates with 1 embryo transferred over 2 cycles vs 2 embryos in 1 cycle. Am J Obstet Gynecol 2015;212:676.e1–7. 10.1016/j.ajog.2015.02.005 25683965 PMC4416976

[38] Crawford S, Boulet SL, Mneimneh AS, Costs of achieving live birth from assisted reproductive technology: a comparison of sequential single and double embryo transfer approaches. Fertil Steril 2016;105:444–50. 10.1016/j.fertnstert.2015.10.032 26604068 PMC5125029

[39] Maheshwari A, Griffiths S, Bhattacharya S. Global variations in the uptake of single embryo transfer. Hum Reprod Update 2011;17:107–20. 10.1093/humupd/dmq028 20634207

[40] Lemos EV, Zhang D, Van Voorhis BJ, Hu XH. Healthcare expenses associated with multiple vs singleton pregnancies in the United States. Am J Obstet Gynecol 2013;209:586.e1–11. 10.1016/j.ajog.2013.10.005 24238479

[41] Grobman WA, Milad MP, Stout J, Klock SC. Patient perceptions of multiple gestations: an assessment of knowledge and risk aversion. Am J Obstet Gynecol 2001;185:920–4. 10.1067/mob.2001.117305 11641679

[42] Blennborn M, Nilsson S, Hillervik C, Hellberg D. The couple’s decision-making in IVF: one or two embryos at transfer? Hum Reprod 2005;20:1292–7. 10.1093/humrep/deh785 15734759

[43] Pinborg A, Loft A, Schmidt L, Andersen AN. Attitudes of IVF/ICSI-twin mothers towards twins and single embryo transfer. Hum Reprod 2003;18:621–7. 10.1093/humrep/deg145 12615836

[44] Ryan GL, Sparks AE, Sipe CS, Syrop CH, Dokras A, Van Voorhis BJ. A mandatory single blastocyst transfer policy with educational campaign in a United States IVF program reduces multiple gestation rates without sacrificing pregnancy rates. Fertil Steril 2007;88:354–60. 10.1016/j.fertnstert.2007.03.001 17490657

[45] Hope N, Rombauts L. Can an educational DVD improve the acceptability of elective single embryo transfer? A randomized controlled study. Fertil Steril 2010;94:489–95. 10.1016/j.fertnstert.2009.03.080 19442970

[46] Sunderam S, Boulet SL, Jamieson DJ, Kissin DM. Effects of patient education on desire for twins and use of elective single embryo transfer procedures during ART treatment: A systematic review. Reprod Biomed Soc Online 2018;6:102–19. 10.1016/j.rbms.2018.10.017 30761357 PMC6287049

[47] Callaghan WM, MacDorman MF, Rasmussen SA, Qin C, Lackritz EM. The contribution of preterm birth to infant mortality rates in the United States. Pediatrics 2006;118:1566–73. 10.1542/peds.2006-0860 17015548

[48] Tanner K, Sabrine N, Wren C. Cardiovascular malformations among preterm infants. Pediatrics 2005;116:e833–8. 10.1542/peds.2005-0397 16322141

[49] Rasmussen SA, Moore CA, Paulozzi LJ, Rhodenhiser EP. Risk for birth defects among premature infants: a population-based study. J Pediatr 2001;138:668–73. 10.1067/mpd.2001.112249 11343041

[50] Waitzman N, Jalali A. Updating national preterm birth costs to 2016 with separate estimates for individual states. Final report to the March of Dimes. https://www.marchofdimes.org/peristats/documents/Cost_of_Prematurity_2019.pdf

[51] D’Angelo DV, Whitehead N, Helms K, Barfield W, Ahluwalia IB. Birth outcomes of intended pregnancies among women who used assisted reproductive technology, ovulation stimulation, or no treatment. Fertil Steril 2011;96:314–320.e2. 10.1016/j.fertnstert.2011.05.073 21718990

[52] Dunietz GL, Holzman C, Zhang Y, Assisted reproductive technology and newborn size in singletons resulting from fresh and cryopreserved embryos transfer. PLoS One 2017;12:e0169869. Erratum in: PLoS One 2018;13:e0196767. 10.1371/journal.pone.0169869 28114395 PMC5256964

[53] Terho AM, Pelkonen S, Opdahl S, High birth weight and large-for-gestational-age in singletons born after frozen compared to fresh embryo transfer, by gestational week: a Nordic register study from the CoNARTaS group. Hum Reprod 2021;36:1083–92. 10.1093/humrep/deaa304 33416878

[54] Kulkarni AD, Jamieson DJ, Jones HW Jr, Fertility treatments and multiple births in the United States. N Engl J Med 2013;369:2218–25. 10.1056/NEJMoa1301467 24304051

[55] Martin JA, Hamilton BE, Osterman MJ. Three decades of twin births in the United States, 1980–2009. NCHS Data Brief 2012;80:1–8. 22617378

[56] Adashi EY, Gutman R. Delayed childbearing as a growing, previously unrecognized contributor to the national plural birth excess. Obstet Gynecol 2018;132:999–1006. 10.1097/AOG.0000000000002853 30204699

[57] Sunderam S, Kissin DM, Crawford SB, Kulkarni AD, Boulet SL. Overview of 2015 U.S. assisted reproductive technology (ART) treatment outcomes and contribution of ART and non-ART fertility treatments to U.S. multiple births. Fertil Steril 2017;108:e97–8. 10.1016/j.fertnstert.2017.07.297

[58] Kulkarni AD, Adashi EY, Jamieson DJ, Crawford SB, Sunderam S, Kissin DM. Affordability of fertility treatments and multiple births in the United States. Paediatr Perinat Epidemiol 2017;31:438–48. 10.1111/ppe.12383 28762537

[59] Barradas DT, Barfield WD, Wright V, D’Angelo D, Manning SE, Schieve LA. Assessment of assisted reproductive technology use questions: Pregnancy Risk Assessment Monitoring System Survey, 2004. Public Health Rep 2012;127:516–23. 10.1177/003335491212700507 22942469 PMC3407851

[60] Sanders J, Simonsen S, Porucznik CA, Baksh L, Stanford JB. Use of fertility treatments in relation to the duration of pregnancy attempt among women who were trying to become pregnant and experienced a live birth. Matern Child Health J 2014;18:258–67. 10.1007/s10995-013-1262-5 23584927

